# Hs1Cas12a and Ev1Cas12a confer efficient genome editing in plants

**DOI:** 10.3389/fgeed.2023.1251903

**Published:** 2023-10-12

**Authors:** Gen Li, Yingxiao Zhang, Micah Dailey, Yiping Qi

**Affiliations:** ^1^ Department of Plant Science and Landscape Architecture, University of Maryland, College Park, College Park, MD, United States; ^2^ Institute for Bioscience and Biotechnology Research, University of Maryland, Rockville, MD, United States

**Keywords:** Cas12a orthologs, Ev1Cas12a, Hs1Cas12a, genome editing, low temperature tolerance, rice, tomato, poplar

## Abstract

Cas12a, also known as Cpf1, is a highly versatile CRISPR-Cas enzyme that has been widely used in genome editing. Unlike its well-known counterpart, Cas9, Cas12a has unique features that make it a highly efficient genome editing tool at AT-rich genomic regions. To enrich the CRISPR-Cas12a plant genome editing toolbox, we explored 17 novel Cas12a orthologs for their genome editing capabilities in plants. Out of them, Ev1Cas12a and Hs1Cas12a showed efficient multiplexed genome editing in rice and tomato protoplasts. Notably, Hs1Cas12a exhibited greater tolerance to lower temperatures. Moreover, Hs1Cas12a generated up to 87.5% biallelic editing in rice T_0_ plants. Both Ev1Cas12a and Hs1Cas12a achieved effective editing in poplar T_0_ plants, with up to 100% of plants edited, albeit with high chimerism. Taken together, the efficient genome editing demonstrated by Ev1Cas12a and Hs1Cas12a in both monocot and dicot plants highlights their potential as promising genome editing tools in plant species and beyond.

## Introduction

Clustered Regularly Interspaced Short Palindromic Repeats (CRISPR)-CRISPR associated protein (Cas) is the predominant RNA-guided nuclease technology for inducing targeted DNA double strand breaks (DSBs) in living organisms. CRISPR-Cas and its derived technologies have been widely used for genome editing, transcriptional regulation, epigenetic modifications, genomic region visualization and isolation, etc (Zhang et al., 2019; Gao, 2021). Cas12a belongs to the Class II Type V CRISPR system and is the second most used CRISPR system in plants (Zetsche et al., 2015; Zhang et al., 2019). Cas12a has a T-rich protospacer adjacent motif (PAM) requirement and generates staggered DSB ends distal from the PAM, resulting in large deletions and high editing efficiency at AT-rich genomic regions. Moreover, Cas12a only requires a short CRISPR RNA (crRNA) for target site recognition, making it an ideal platform for multiplexed genetic engineering and ribonucleoprotein (RNP) delivery (Zetsche et al., 2015; Zhang et al., 2022).

CRISPR-Cas12a is a powerful tool for genome editing in many plant species (Zhang et al., 2019; Bandyopadhyay et al., 2020; Schindele et al., 2020). Recently, an efficient Cas12a-based promoter editing system has been developed to introduce quantitative traits in crops (Zhou et al., 2023). So far, multiple Cas12a nucleases have been demonstrated in plants, including AsCas12a, LbCas12a (Tang et al., 2017), FnCas12a (Zhong et al., 2018), ErCas12a (also known as MAD7) (Lin et al., 2021), and Mb2Cas12a (Zhang et al., 2021b). Moreover, researchers have improved genome editing efficiency of Cas12a through protein engineering, such as LbCas12a-D156R/ttLbCas12a (Schindele and Puchta, 2020), AsCas12a ultra (Zhang et al., 2021a), intron-containing ttLbCas12a (Schindele et al., 2023), LbCas12a-RV and LbCas12a-RRV (Zhang et al., 2023). Nevertheless, the temperature sensitivity of Cas12a represents a constraint that affects Cas12-mediated genome editing (Malzahn et al., 2019; Zhang et al., 2021b). Given that a significant proportion of plant species are transformed under ambient temperatures, it becomes imperative to identify new CRISPR-Cas12a systems that confer robust genome editing efficiency under lower temperatures.

To broaden the application of the CRISPR-Cas12a system, seventeen novel Cas12a orthologs have been identified and investigated in this study. To fully compare their performance in genome editing, these novel Cas12a orthologs were tested in various plant species and plant systems (protoplasts and stable transgenic lines), as well as at different temperatures. Our results demonstrated that Ev1Cas12a and Hs1Cas12a enable efficient genome editing in various plant species and hold great potential as robust genome editing tools in a wider range of plant species.

## Results

### Identification and selection of new Cas12a orthologs

**FIGURE 1:**
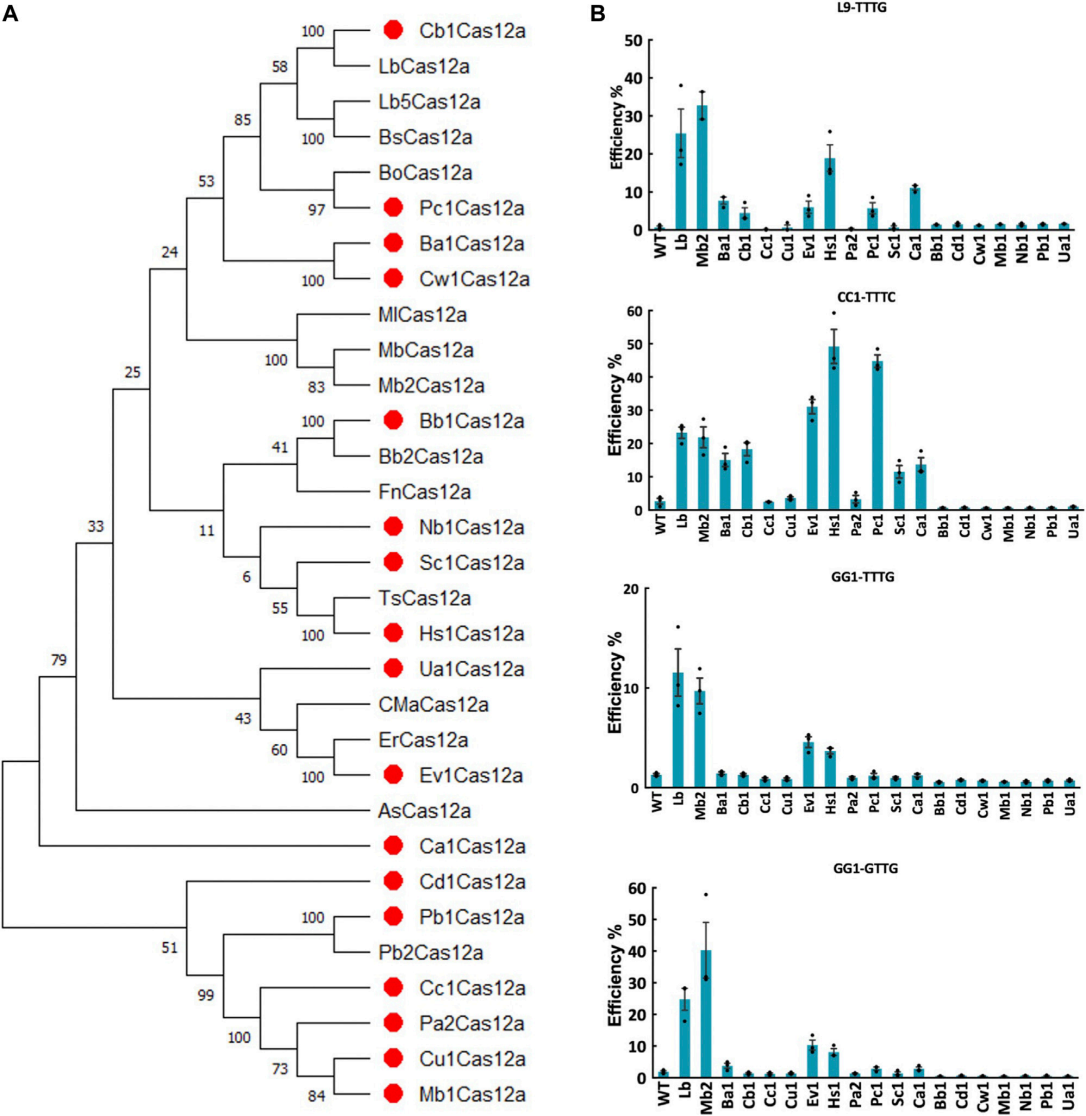
Screening of novel Cas12a orthologs in rice protoplasts. **(A)**, phylogenetic tree of Cas12a nucleases using MEGA11. Amino acid sequences of Cas12a ortholog candidates was acquired from Joint Genome Institute Microbial Genomes and Microbiomes (JGI IMG/M) database by using LbCas12a, FnCas12a, ErCas12a (MAD7) and Mb2Cas12a as the BLAST queries. Seventeen novel Cas12a nuclease candidates were labeled with red dots. **(B)**, targeted mutagenesis efficiencies (percentage) of 17 novel Cas12a orthologs at four target sites with TTTV and VTTV PAMs in rice protoplasts. WT, protoplasts transformed with water. LbCas12a and Mb2Cas12a were used as controls. Data are presented as mean values ±SEM. *n* = 3 biologically independent samples.

Since genome editing in plants is mostly conducted at ambient temperatures, we are interested in identifying Cas12a nucleases that are more tolerant to lower temperatures. We identified new Cas12a ortholog candidates using LbCas12a, FnCas12a, ErCas12a, Mb2Cas12a as queries to BLAST (Basic Local Alignment Search Tool) against the Joint Genome Institute Microbial Genomes and Microbiomes database, and selected Cas12a nucleases originated from bacteria with ambient living temperatures and capability to reproduce in soil. The novel Cas12a nucleases all have estimated length of more than 1,000 amino acids and less than 80% sequence homology to queries (Figure 1A; Supplementary Figure S1). Finally, seventeen Cas12a orthologs were identified and selected to test in plants, including Ba1Cas12a, Ca1Cas12a, Cb1Cas12a, Cc1Cas12a, Cu1Cas12a, Ev1Cas12a, Hs1Cas12a, Pa2Cas12a, Pc1Cas12a, Sc1Cas12a, Bb1Cas12a, Cd1Cas12a, Cw1Cas12a, Mb1Cas12a, Nb1Cas12a, Pb1Cas12a and Ua1Cas12a (Figure 1A; Supplementary Table S1).

To test the editing efficiency of these 17 Cas12a orthologs, we first conducted rice protoplast transformation targeting four sites with TTTV and VTTV PAMs at 32°C. Screening of 17 Cas12a orthologs showed variable genome editing activities across these target sites. Seven Cas12a nucleases (Ba1Cas12a, Cb1Cas12a, Ev1Cas12a, Hs1Cas12a, Pc1Cas12a, Sc1Cas12a and Ca1Cas12a) showed comparable or higher genome editing efficiencies than LbCas12a and Mb2Cas12a at the CC1-TTTC target site (Figure 1B). At this site, the average editing efficiencies for Ev1Cas12a, Hs1Cas12a and Pc1Cas12a reached 31.05%, 49.17% and 44.76%, respectively, which were higher than LbCas12a and Mb2Cas12a (Figure 1B). Moreover, Ev1Cas12a and Hs1Cas12a were able to reliably edit both TTTV and VTTV PAM sites (Figure 1B). Taken together, Ev1Cas12a and Hs1Cas12a stood out in this screening and were selected for further studies.

### Genome editing of Ev1Cas12a and Hs1Cas12a in rice protoplasts at low temperatures

**FIGURE 2:**
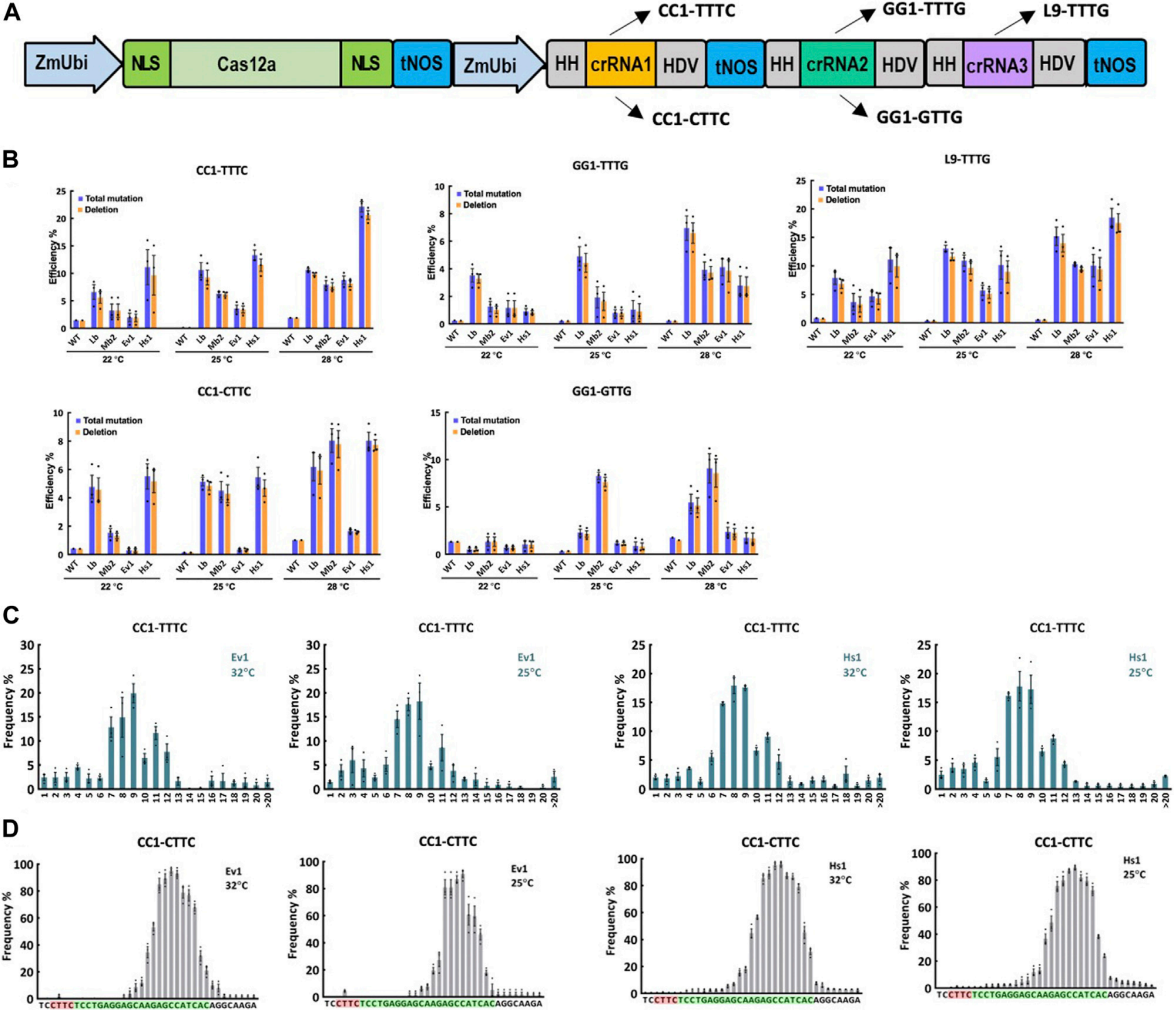
Genome editing of Ev1Cas12a and Hs1Cas12a in rice protoplasts at low temperatures. **(A)**, multiplexed genome editing of three crRNAs to target five sites using a dual ZmUbi promoter and tandem HH-crRNA-HDV system. **(B)**, total mutation and deletion efficiencies (percentage) of Ev1Cas12a and Hs1Cas12a at five target sites with TTTV and VTTV PAMs at 28°C, 25°C, and 22°C in rice protoplasts compared with LbCas12a and Mb2Cas12a. WT, protoplasts transformed with water. **(C)**, deletion size of Ev1Cas12a and Hs1Cas12a at CC1-TTTC site in rice protoplasts. **(D)**, deletion position of Ev1Cas12a and Hs1Cas12a at CC1-CTTC site in rice protoplasts. PAM sequence is highlighted in red and protospacer sequence is highlighted in green. Data are presented as mean values ±SEM. *n* = 3 biologically independent samples.

To further investigate the editing activity of Ev1Cas12a and Hs1Cas12a at lower temperatures, including 22°C, 25°C, and 28°C, multiplexed genome editing was performed with Ev1Cas12a and Hs1Cas12a comparing to LbCas12a and Mb2Cas12a in rice protoplasts (Figure 2A). Three crRNAs were used to target five sites L9-TTTG, CC1-CTTC, CC1-TTTC, GG1-GTTG and GG1-TTTG (Figure 2A). At L9-TTTG, CC1-TTTC and CC1-CTTC sites, Hs1Cas12a showed higher or comparable editing efficiencies when compared with LbCas12a and Mb2Cas12a (Figure 2B). Moreover, Hs1Cas12a showed remarkably higher editing efficiencies than Ev1Cas12a regardless of the temperature (Figure 2B), which indicates Hs1Cas12a has low temperature sensitivity in rice protoplasts. For the low-activity sites GG1-GTTG and GG1-TTTG, both Ev1Cas12a and Hs1Cas12a showed comparable editing efficiency, albeit lower than the other three sites (Figure 2B). In summary, Hs1Cas12a showed remarkable editing efficiency at various low temperatures in rice protoplasts.

### Editing profiles of Ev1Cas12a and Hs1Cas12a in rice protoplasts

The analysis of NGS data revealed the editing profiles of these two novel Cas12a orthologs. As expected, the majority of edits were deletions (Figure 2B). Deletion sizes ranged from 4 to 12 bp for both Cas12a orthologs (Figure 2C; Supplementary Figure S2), which is consistent with other Cas12a nucleases previously described (Tang et al., 2017; Zhong et al., 2018; Zhang et al., 2021b). Deletion sizes were not affected by temperature significantly. Both Ev1Cas12a and Hs1Cas12a showed similar deletion position profiles (12–23 bp from PAM), which are not affected by temperature (Figure 2D; Supplementary Figure S3). These data suggest both Ev1Cas12a and Hs1Cas12a cleave target sites in a similar fashion, creating staggered DNA DSBs at the PAM-distal sites.

### Genome editing of three novel Cas12a orthologs in tomato protoplasts

**FIGURE 3:**
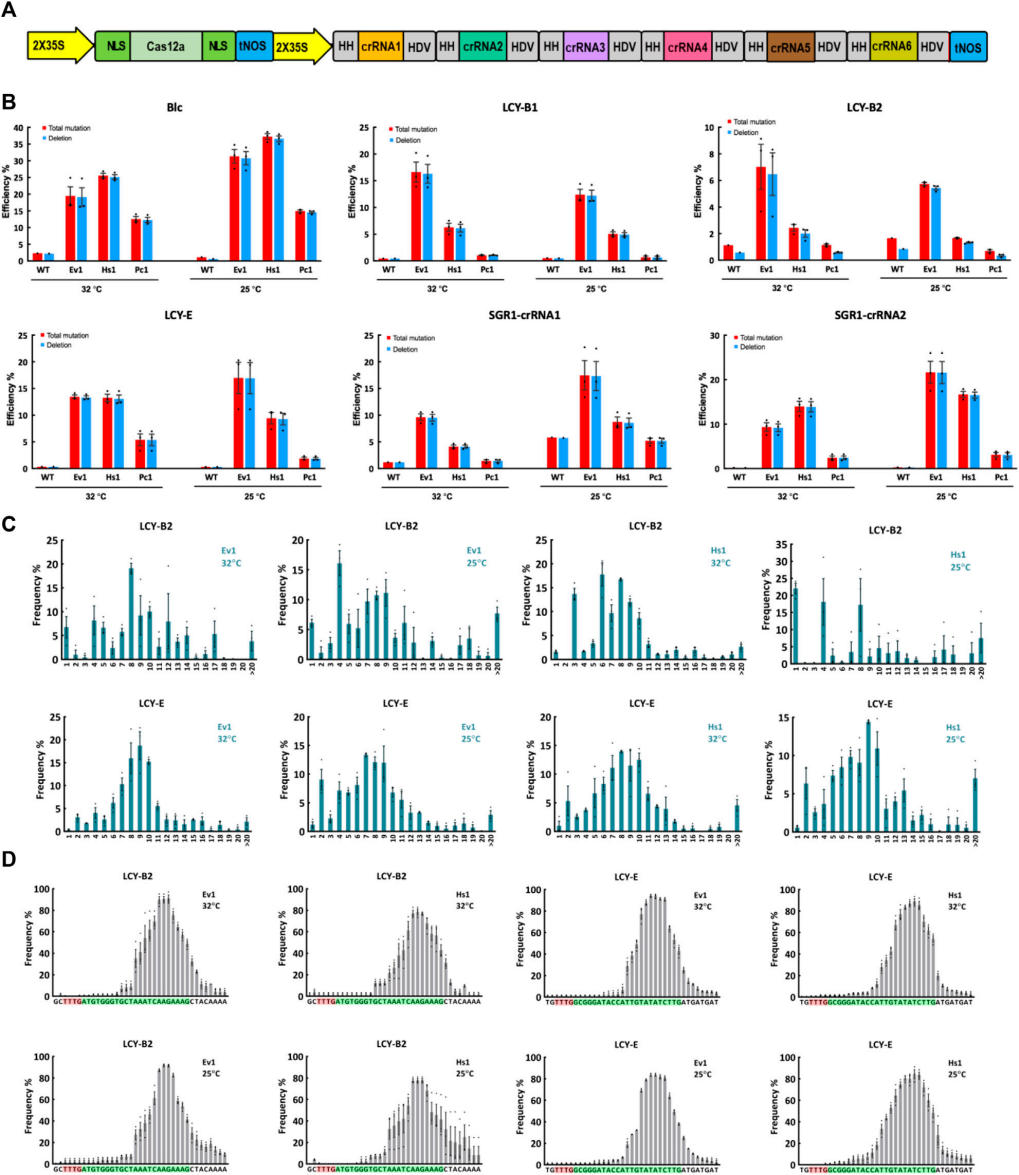
Genome editing of Ev1Cas12a, Hs1Cas12a and Pc1Cas12a orthologs in tomato protoplasts. **(A)**, multiplexed genome editing of six crRNA in tomato using a dual 2 × 35S promoter and tandem HH-crRNA-HDV system. **(B)**, total mutation and deletion efficiencies (percentage) of two novel Cas12a orthologs at six target sites with TTTV PAMs at 25°C and 32°C in tomato protoplasts. WT, protoplasts transformed with water. **(C)**, deletion size of Hs1Cas12a and Ev1Cas12a at two target sites in tomato protoplasts. **(D)**, deletion position of Hs1Cas12a and Ev1Cas12a at two target sites in tomato protoplasts. PAM sequence is highlighted in red and protospacer sequence is highlighted in green. Data are presented as mean values ±SEM. *n* = 3 biologically independent samples.

We then tested editing performance of Ev1Cas12a, Hs1Cas12a and Pc1Cas12a in a dicot crop, tomato. Since Pc1Cas12a showed comparable editing efficiency to Ev1Cas12a and Hs1Cas12a at L9-TTTV and CC1-TTTC sites in rice protoplasts (Figure 1B), we included it here for testing in a dicot plant. Based on our previously developed Cas12a multiplexed editing system (Zhang et al., 2021b), we conducted multiplexed genome editing by targeting six sites with TTTV PAMs in tomato protoplasts (Figure 3A). Amplicon sequencing using NGS revealed efficient genome editing by Ev1Cas12a and Hs1Cas12a at most target sites (Figure 3B). However, Pc1Cas12a only showed efficient editing at the *Blc* site (Figure 3B). Remarkably, Ev1Cas12a showed higher editing efficiencies at 25°C than at 32°C at *SGR1*-crRNA1, *SGR1*-crRNA2, *Blc,* and *LCY-E* sites. Similar trends were observed on Hs1Cas12a at *SGR1*-crRNA1, *SGR1*-crRNA2, and *Blc* sites. Taken together, both Ev1Cas12a and Hs1Cas12a showed remarkable editing efficiency and low temperature tolerance in tomato protoplasts.

### Editing profiles of Ev1Cas12a and Hs1Cas12a in tomato protoplasts

Similar to what was observed in rice, deletion is the predominant edit type in tomato protoplasts (Figures 2B, 3B). Deletion sizes ranged from 3 to 13 bp and were not affected by temperature significantly (Figure 3C; Supplementary Figure S4). Deletion positions were about 12–23 bp away from the PAM and not affected by temperature significantly as well (Figure 3D). Collectively, these results indicate Ev1Cas12a and Hs1Cas12a generate similar editing profiles in different plant species.

### Genome editing of Hs1Cas12a in transgenic rice plants

**FIGURE 4:**
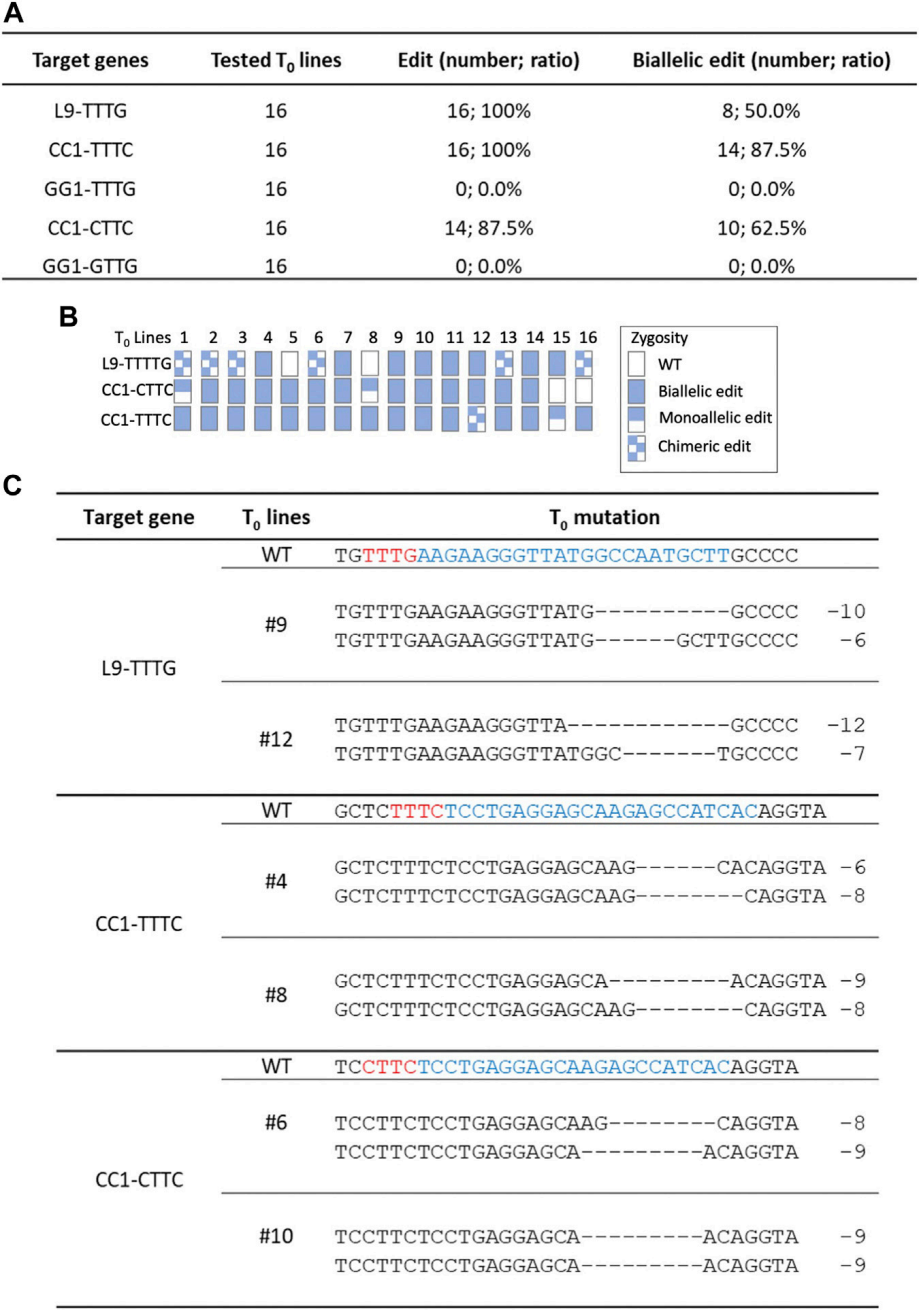
Genome editing of Hs1Cas12a in rice T_0_ transgenic plants. **(A)**, editing efficiency of Hs1-Cas12a at five target sites in rice T_0_ plants. **(B)**, genotypes of 16 T_0_ transgenic plants. **(C)**, examples of mutation profiles for biallelic mutant lines at three target sites. Sequences in blue indicate crRNA target sites and in red indicate PAM sequences.

Since Hs1Cas12a showed significant editing efficiency in rice protoplasts, its editing efficiency was further tested in rice transgenic plants. Three crRNAs were multiplexed to target five sites L9-TTTG, CC1-TTTC, CC1-CTTC, GG1-TTTG and GG1-GTTG (Figure 2A), and 16 T_0_ plants were generated for genotyping. The total editing efficiency and biallelic editing efficiency was 87.5%–100% and 50%–87.5%, respectively at three target sites L9-TTTG, CC1-TTTC and CC1-CTTC (Figures 4A, B). No significant editing was detected at GG1-TTTG and GG1-GTTG sites (Figure 4A), which was consistent with the low editing activity (<4%) that was previously observed at these two sites in rice protoplasts (Figure 2B). In the biallelic edited T_0_ plants, the deletion range was 2–12 bp at L9-TTTG, CC1-TTTC, and CC1-CTTC sites (Figure 4C; Supplementary Figure S5), which is similar to deletion sizes observed in rice protoplasts (Figure 2C; Supplementary Figure S2). Therefore, Hs1Cas12a enables highly efficient genome editing in stable rice plants.

### Genome editing of Hs1Cas12a and Ev1Cas12a in transgenic poplar plants

**FIGURE 5:**
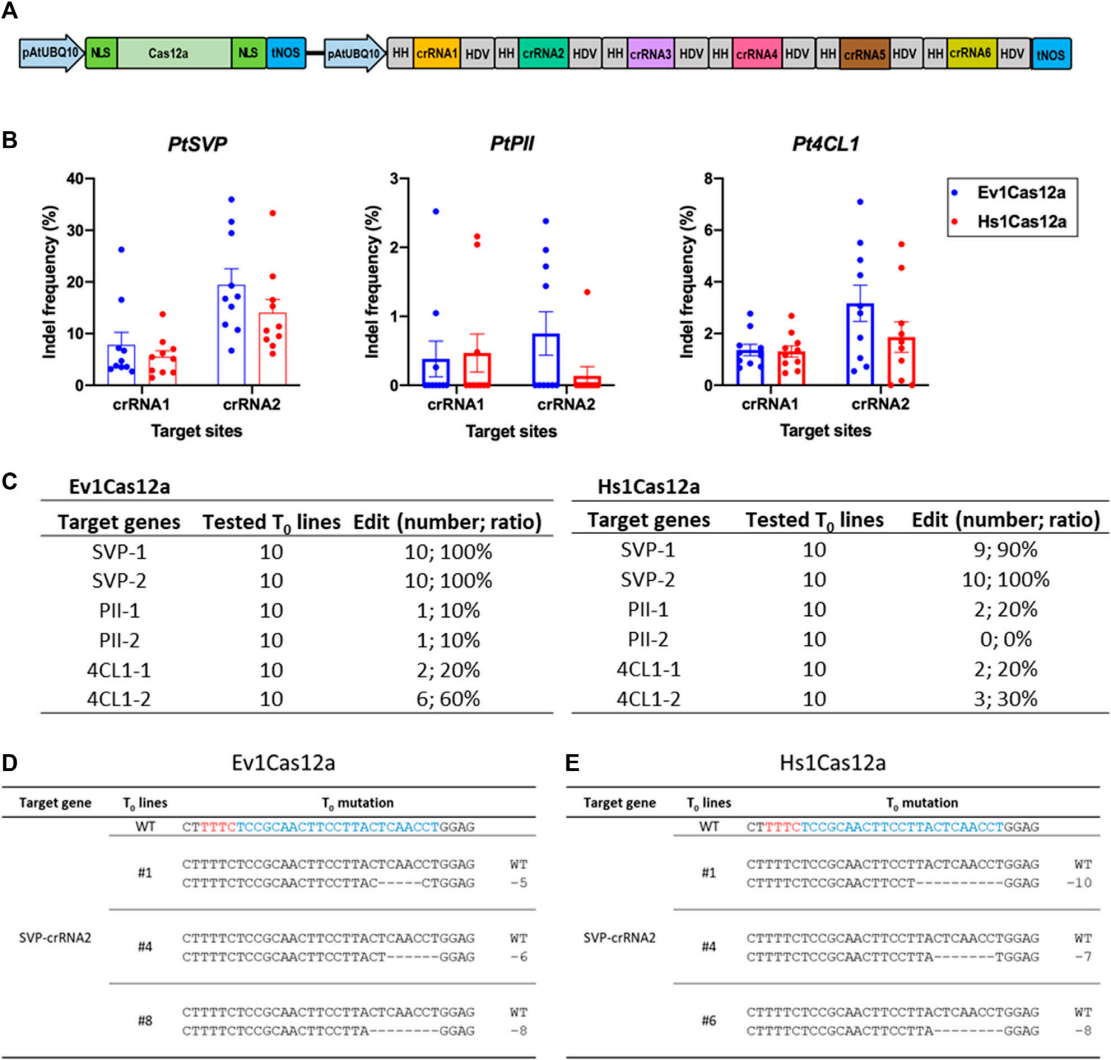
Genome editing of Hs1Cas12a and Ev1Cas12a in poplar T_0_ transgenic plants. **(A)**, multiplexed genome editing of six crRNAs in poplar using a dual AtUBQ10 promoter and tandem HH-crRNA-HDV system. **(B)**, indel frequency of Hs1Cas12a and Ev1Cas12a at six target sites in poplar T0 plants. **(C)**, editing efficiency of Hs1Cas12a and Ev1Cas12a at six target sites in T_0_ plants. T_0_ transgenic plants with 2% or higher indel frequency was counted as edited plants. **(D)**, examples of mutation profiles for T_0_ lines at SVP-crRNA2 target site by Ev1Cas12a. **(E)**, examples of mutation profiles for T_0_ lines at SVP-crRNA2 target site by Hs1Cas12a. Sequences in blue indicate crRNA target sites and in red indicate PAM sequences.

We next decided to further test the genome editing capability of Hs1Cas12a and Ev1Cas12a in another dicot species, poplar, a woody plant model and a bioenergy crop. Six crRNAs were multiplexed to target three genes *4CL1, PII* and *SVP* in poplar (Figure 5A). Ten T_0_ plants were tested for each transformation. Ev1Cas12a and Hs1Cas12a showed up to 19.5% and 14.0% insertion and deletion (indel) frequencies at two SVP target sites, respectively (Figure 5B). Both Ev1Cas12a and Hs1Cas12a showed low indel frequencies at the other four target sites (4CL1-1, 4CL1-2, PII-1, and PII-2), for which low indel frequencies were also observed for LbCas12a and AsCas12a in our pervious study (Zhang et al., 2023). The percentages of edited plants (indel frequency >2%) by Ev1Cas12a and Hs1Cas12a at all six target sites is 10%–100% and 0%–100%, respectively (Figure 5C), while no biallelic editing was detected (Figures 5D, E). Thus, both Hs1Cas12a and Ev1Cas12a enable somatic genome editing in poplar transgenic lines.

### Off-target analysis of Hs1Cas12a and Ev1Cas12a in transgenic poplar plants

Previous studies showed Cas12a orthologs and engineered Cas12a had low off-target effects (Zhang et al., 2021b; Zhang et al., 2023). To further verify this feature of Cas12a, we analyzed off-target effects of both Ev1Cas12a and Hs1Cas12a in poplar T_0_ plants. Three top off-target sites were selected for each of two high-activity crRNAs, SVP-crRNA1 and SVP-crRNA2, using Cas-OFFinder. Editing frequency at off-target sites was analyzed using NGS of PCR amplicons. As shown in Supplementary Figure S6, there is no editing at five off-target sites (OT1-OT5) in WT plants. For the OT-6 site, low frequency mutations (∼6–7%) were observed in the WT as well as the EvCas12a and Hs1Cas12a samples, suggesting there was an elevated level of background mutations at this site regardless of sample types. Therefore, our results demonstrate that Ev1Cas12a and Hs1Cas12a have high specificity for multiplexed genome editing in plants.

## Discussion

Although CRISPR-Cas9 mediated genome editing is predominantly applied in plants, the use of CRISPR-Cas12a is increasing manifestly for genome engineering due to its versatile and simplified features, including preferring T-rich target regions, a small guide RNA devoid of trans-activating CRISPR RNA (tracrRNA), simpler multiplexing, and generating double strand break with staggered ends (Zhang et al., 2019; Bandyopadhyay et al., 2020). In the past few years, several Cas12a nucleases have been identified and used for genome editing in plants, including AsCas12a (Tang et al., 2017), LbCas12a (Hu et al., 2017; Wang et al., 2017), FnCas12a (Endo et al., 2016), ErCas12a or MAD7 (Lin et al., 2021), Mb2Cas12a, MbCas12a, ErCas12a, Lb5Cas12a, BsCas12a, and TsCas12a (Zhang et al., 2021b). There is still a great necessity to further develop the Cas12a system and achieve editing stability especially at ambient temperatures in diverse plant species. To accomplish this goal, seventeen Cas12a orthologs were identified for testing in this study. Our screening of editing activity focused on rice first and followed up the promising ones in tomato and poplar. In the first screening in rice protoplasts, seven out of 17 Cas12a nucleases showed detectable editing efficiency (Figure 1B). In this study, all 17 new Cas12a orthologs used the Fn crRNA scaffold and the predicted crRNAs for some novel Cas12a orthologs are distinct from the FnCas12a crRNA (Supplementary Figure S7). The crRNA scaffolds of Mb2Cas12a and FnCas12a are very similar. When we pair FnCas12a′s crRNA scaffold with Mb2Cas12a, it resulted in similar editing efficiency to MbCas12a pairing with its own crRNA scaffold, based on testing at six target sites in rice protoplasts (Supplementary Figure S8). Consistent with this, the crRNA scaffolds of Ev1Cas12a, Hs1Cas12a, and Pc1Cas12a, which showed top editing activities among all 17 new Cas12a orthologs tested, resemble that of FnCas12a (Supplementary Figure S7). It is possible some of the tested Cas12a orthologs that failed to show genome editing activity in our screen might display some editing activity when paired with their own crRNA scaffolds. This warrants future investigation.

Hs1Cas12a gave comparable and even higher editing efficiency than LbCas12a and Mb2Cas12a at CC1-TTTC and L9-TTTG at 32°C (Figure 1B). Previously, we reported that temperature could affect editing activity of Cas12a and editing activity was barely observed at 22°C in both *Arabidopsis* and maize (Malzahn et al., 2019). However, Hs1Cas12a showed manifest editing efficiency at CC1-TTTC and L9-TTTC sites at 22°C (Figure 2B), which indicates Hs1Cas12a may achieve high editing efficiency in plants at lower temperatures. In contrast to rice protoplasts, higher editing efficiency was observed for Ev1Cas12a than for Hs1Cas12a in tomato protoplasts. This distinct performance is not unexpected, since AsCas12a enables high editing efficiency in human cells and poplar but low efficiency in rice (Zetsche et al., 2015; Tang et al., 2017; An et al., 2020). Together, our data, along with these earlier publications, suggest different Cas12a orthologs may have different genome editing activities in different plant species. It justifies the importance of discovering more Cas12a orthologs for genome editing as done in this study.

In this work, we have explored 17 novel Cas12a orthologs and demonstrated that Hs1Cas12a and Ev1Cas12a enable efficient genome editing in rice, tomato, and poplar. Cas12a orthologs and engineered Cas12a showed low off-target effects (Zhang et al., 2021b; Zhang et al., 2023). Consistently, our two Cas12a orthologs Ev1Cas12a and Hs1Cas12a exhibit minimal crRNA-dependent off-target effects (Supplementary Figure S6). The background mutations that we detected on one off-target site (OT6) may be caused by tissue culture process as reported previously (Tang et al., 2018). Taken together, our work has demonstrated new Cas12a nucleases for highly efficient genome editing in different plant species at different temperatures. We anticipate further improvement of these new Cas12a orthologs via protein engineering. We and others have previously shown that protein engineering can substantially improve Cas12a genome editing efficiency, temperature sensitivity and PAM requirements (Gao et al., 2017; Zhong et al., 2018; Kleinstiver et al., 2019; Schindele and Puchta, 2020; Zhang et al., 2021a; Schindele et al., 2023; Zhang et al., 2023). We envision that these same protein engineering principles could be applied to further boost the genome editing performance of our Cas12a ortholog collection. Hence, our study here provides new Cas12a resources to expand CRISPR application for plant breeding and crop improvement in a broader range of plant species. Needleless to say, these Cas12a orthologs and their future improved versions would also allow for efficient genome editing in other non-plant organisms including animals and humans.

## Materials and methods

### Identification and selection of novel Cas12a orthologs

Cas12a ortholog candidates were identified by performing BLAST searches against the Joint Genome Institute Microbial Genomes and Microbiomes (JGI IMG/M) database, using LbCas12a, FnCas12a, ErCas12a, and Mb2Cas12a as queries. Cas12a nucleases derived from bacteria with ambient living temperature and soil reproductive capability were specifically chosen. The novel Cas12a orthologs were identified based on their estimated length of over 1,000 amino acids and less than 80% sequence homology to the query sequences. A phylogenetic tree was constructed using the Neighbor-Joining method with MEGA11. Ultimately, 17 Cas12a orthologs were selected for genome editing evaluation, namely, Ba1Cas12a, Ca1Cas12a, Cb1Cas12a, Cc1Cas12a, Cu1Cas12a, Ev1Cas12a, Hs1Cas12a, Pa2Cas12a, Pc1Cas12a, Sc1Cas12a, Bb1Cas12a, Cd1Cas12a, Cw1Cas12a, Mb1Cas12a, NbCas12a, PbCas12a, and UaCas12a (Supplementary Table S1).

### Vector construction

To assess the genome editing efficiency of all 17 novel Cas12a orthologs in rice, Cas12a genes were synthesized with rice codon optimization and cloned into pYPQ230 (Addgene #86210, Tang et al., 2017) at the NotI and NcoI sites to construct the Cas12a entry clones. Three crRNAs were used to target five sites: L9 targeting OsEPFL9 with the TTTV PAM; CC1 targeting Os02g46610 with the TTV PAM and Os04g50120 with the TTTV PAM; GG1 targeting Os12g24050 with the TTV PAM and Os01g23900 with the TTTV PAM. For single crRNA cloning, the crRNA was cloned into pYPQ141-ZmUbi-RZ-Fn (Addgene #108864, Zhong et al., 2018) at the BsmBI site as previously described to construct the crRNA entry clones (Zhong et al., 2018) (Supplementary Table S2). Cas12a entry clones and crRNA entry clones were assembled with the destination vector pYPQ203 (Addgene #86207, Tang et al., 2017) by Gateway LR reactions to generate the final T-DNA vectors (Supplementary Table S3). LbCas12a and Mb2Cas12a were used as controls. pYPQ141-ZmUbi-RZ-Lb (Addgene #86197, Tang et al., 2017) was used for crRNA cloning of LbCas12a.

To assess the genome editing of three novel Cas12a orthologs (Ev1Cas12, Hs1Cas12a and Pc1Cas12a) in tomato, the same Cas12a entry clones for rice genome editing were used. A multiplexed crRNA entry clone was generated containing six crRNAs targeting five genes involved in lycopene synthesis and catabolism, including *SGR1* (two crRNAs), *lycopene ε-cyclase* (*LCY-E*), *beta-lycopene cyclase* (*Blc*), *lycopene β-cyclase1* (*LCY-B1*), and *lycopene β-cyclase2* (*LCY-B2*). A tandem ribozyme multiplexing system was used as previously described (Zhang et al., 2021b). The 2 × 35S promoter was used for crRNA expression. Cas12a entry clones and the multiplexed crRNA entry clone were assembled with the destination vector pMDC32 by Gateway LR reactions to form the final T-DNA vectors (Supplementary Tables S2, S3).

Similarly in poplar, a multiplexed six crRNAs entry clone was generated to target three genes, *Pt4CL1*, *PtPII,* and *PtSVP,* using the tandem ribozyme multiplexing system as described previously (Zhang et al., 2021b). Cas12a entry clones and crRNA entry clones were then assembled with the destination vector pYPQ202 (Addgene #86198) using Gateway LR reactions to generate the final T-DNA vectors (Supplementary Tables S2, S3).

### Rice and tomato protoplast transformation

Rice protoplasts were isolated and transformed as previously described (Tang et al., 2016). Briefly, rice seedlings (Japonica cultivar Kitaake) were grown in the dark for 14 days and cut into 0.5–1.0 mm strips and placed in the enzyme solution. After incubation at 28°C for 8 h in dark, rice protoplasts were filtered through a 75 μm cell strainer and washed with W5 buffer followed by sucrose density gradient centrifugation to enrich viable protoplast. After cell counts, protoplasts were resuspended in the MMG buffer and the concentration was adjusted to 2 × 10^6^ cells/mL. Two hundred μL protoplasts were mixed with 30 μg (30 μL) plasmid, and then mixed with 230 μL polyethylene glycol (40% PEG). The mixture was set at room temperature for 30 min and then 900 μL W5 were added to stop the transformation. Protoplasts were collected and resuspended in 1 mL W5 buffer and incubated in 24-well culture plates at 32°C (or 22°C, 25°C, 28°C according to the experiments) in dark for 2 days. Protoplasts transformed with water were used as the wild type (WT) control for all genome editing experiments.

Tomato seedlings (M82) were grown with 16h/8 h light/dark photoperiod for 7–9 days until the cotyledons were fully expanded. Cotyledons were cut off and placed in the same enzyme solution used for rice. After incubation at 28°C for 8 h in dark, tomato protoplasts were filtered through a 75 μm cell strainer and collected by centrifugation. To purify the viable protoplast, 6 mL 0.55 M sucrose solution was added to the protoplasts followed by 2 mL W5 on top. After centrifuge for 30 min at 200 g, protoplasts were collected at the interface. Protoplasts were moved to new tubes and washed with W5 buffer twice. Protoplasts were then resuspended in the MMG buffer and used for transformation based on the same method for rice.

### Rice and poplar stable transformation

For the transformation of the rice plants, *Agrobacterium*-mediated transformation was carried out following the previously described method (Zhang et al., 2021b). The regenerated shoots were then cultured at 29°C under 16 h of light and 8 h of darkness. Genomic DNA was extracted from young leaves of T_0_ plants using a CTAB method (Clarke, 2009).

To perform stable transformation in poplar, an improved *Agrobacterium*-mediated transformation method (Li et al., 2023) was used. Regenerated shoots were transferred to rooting medium after being selected on shoot induction medium containing hygromycin. The rooted T_0_ plants were propagated and cultured under 16 h of light and 8 h of darkness at 25°C. Young leaves of T_0_ plants were used for DNA extraction followed by genome editing assessment.

### Genome editing efficiency assessment

Protoplast were lysed and target sites were amplified using the Phire Plant Direct PCR Kit (Thermo Scientific). For high-throughput next-generation sequencing (NGS), target sites were amplified with two rounds of PCR using barcoded primers (Supplementary Table S4). Pooled amplicons were subjected for sequencing using the HiSeq2500 platform (Azenta). Editing efficiencies, deletion size and deletion position profiles were analyzed using CRISPRMatch (You et al., 2018).

## Data Availability

The datasets presented in this study can be found in online repositories. The names of the repository/repositories and accession number(s) can be found below: https://www.ncbi.nlm.nih.gov/, PRJNA972439.
