# Supplementary material for: Hs1Cas12a and Ev1Cas12a confer efficient genome editing in plants
Source: Front Genome Ed. 2023 Oct 12;5:1251903. doi: 10.3389/fgeed.2023.1251903 (PMC10602648; doi:10.3389/fgeed.2023.1251903)
Supplement: Supplementary file 1 [file Table1.DOCX]

Table S1. Bacteria names of new Cas12a orthologs

| Cas12a orthologs | Bacteria name |
| --- | --- |
| Ba1Cas12a | Brumimicrobium aurantiacum |
| Ca1Cas12a | Catenovulum sp. CCB-QB4 |
| Cb1Cas12a | Clostridiales bacterium RUG149 |
| Cc1Cas12a | Candidatus Campbellbacteria bacterium RIFCSPLOWO2_01_FULL_34_15 |
| Cu1Cas12a | Candidatus Uhrbacteria bacterium CG11_big_fil_rev_8_21_14_0_20_41_9 |
| Ev1Cas12a | Eubacterium ventriosum |
| Hs1Cas12a | Hydrogenovibrio sp. XS5 |
| Pa2Cas12a | Parcubacteria bacterium JGI MDM2 000213CP-K14 |
| Pc1Cas12a | Porphyromonas cansulci JCM 13913 |
| Sc1Cas12a | Sedimentisphaera cyanobacteriorum |
| Bb1Cas12a | Bdellovibrionales bacterium |
| Cd1Cas12a | candidate division WWE3 bacterium CG10_big_fil_rev_8_21_14_0_10_35_32 |
| Cw1Cas12a | Candidatus Woesearchaeota archaeon CG10_big_fil_rev_8_21_14_0_10_37_12 |
| Mb1Cas12a | Microgenomates (Roizmanbacteria) bacterium GW2011_GWA2_37_7 |
| Nb1Cas12a | Nitrospinae bacterium RIFCSPLOWO2_02_FULL_39_110 |
| Pb1Cas12a | Planctomycetes bacterium GWC2_39_26 |
| Ua1Cas12a | Unclassified Actinobacteria Nt197P3bin131 |

Table S2. crRNAs used in this study

| **Target site** | **PAM** | **Protospacer sequence** |
| --- | --- | --- |
| L9 | TTTG | AAGAAGGGTTATGGCCAATGCTT |
| CC1-TTTC | TTTC | TCCTGAGGAGCAAGAGCCATCAC |
| GG1-TTTG | TTTG | AGCATATGGTTGTAACTTCAGAA |
| CC1-CTTC | CTTC | TCCTGAGGAGCAAGAGCCATCAC |
| GG1-GTTG | GTTG | AGCATATGGTTGTAACTTCAGAA |
| Pt4CL1-crRNA1 | TTTG | TTCATCAGGGACTACAGGGTTGC |
| Pt4CL1-crRNA2 | TTTG | CCAAGGAACCATTCGACATAAAA |
| PtPII-crRNA1 | TTTG | AAACCTCTCAAGTTGAAGAATTC |
| PtPII-crRNA2 | TTTG | AGGCTTTGGGGCTCAAGGTGGTT |
| PtSVP-crRNA1 | TTTG | CACTCGAAGAATCTTGAGAAGCT |
| PtSVP-crRNA2 | TTTC | AGGTTGAGTAAGGAAGTTGCGGA |
| SGR1-1 | TTTG | GCTATCTCCCAAACCATCAATAA |
| SGR1-2 | TTTG | CTAGACTCAGAAACTACATCTTC |
| BLC | TTTG | TGTTAAAGCTAGGAGTAGTACTC |
| LCYB1 | TTTG | GGTAGAAGTGTTTGTGTTAAGGG |
| LCYB2 | TTTG | ATGTGGGTGCTAAATCAAGAAAG |
| LCYE | TTTG | GCGGGATACCATTGTATATCTTG |

Table S3. T-DNA vectors used in this study

| **Vector #** | **Target site** | **crRNA clone** | **Cas12a** | **Expression vector** |
| --- | --- | --- | --- | --- |
| 3365 | L9 | pYPQ141-ZmUbi-RZ-L9 | Ba1Cas12 | pYPQ203 |
| 3366 | L9 | pYPQ141-ZmUbi-RZ-L9 | Ca1Cas12a | pYPQ203 |
| 3367 | L9 | pYPQ141-ZmUbi-RZ-L9 | Cb1Cas12a | pYPQ203 |
| 3368 | L9 | pYPQ141-ZmUbi-RZ-L9 | Cc1Cas12a | pYPQ203 |
| 3369 | L9 | pYPQ141-ZmUbi-RZ-L9 | Cu1Cas12a | pYPQ203 |
| 3370 | L9 | pYPQ141-ZmUbi-RZ-L9 | Ev1Cas12a | pYPQ203 |
| 3371 | L9 | pYPQ141-ZmUbi-RZ-L9 | Hs1Cas12a | pYPQ203 |
| 3372 | L9 | pYPQ141-ZmUbi-RZ-L9 | Pa2Cas12a | pYPQ203 |
| 3373 | L9 | pYPQ141-ZmUbi-RZ-L9 | Pc1Cas12a | pYPQ203 |
| 3374 | L9 | pYPQ141-ZmUbi-RZ-L9 | Sc1Cas12a | pYPQ203 |
| 3375 | CG2 | pYPQ141-ZmUbi-RZ-CG2 | Ba1Cas12 | pYPQ203 |
| 3376 | CG2 | pYPQ141-ZmUbi-RZ-CG2 | Ca1Cas12a | pYPQ203 |
| 3377 | CG2 | pYPQ141-ZmUbi-RZ-CG2 | Cb1Cas12a | pYPQ203 |
| 3378 | CG2 | pYPQ141-ZmUbi-RZ-CG2 | Cc1Cas12a | pYPQ203 |
| 3379 | CG2 | pYPQ141-ZmUbi-RZ-CG2 | Cu1Cas12a | pYPQ203 |
| 3380 | CG2 | pYPQ141-ZmUbi-RZ-CG2 | Ev1Cas12a | pYPQ203 |
| 3381 | CG2 | pYPQ141-ZmUbi-RZ-CG2 | Hs1Cas12a | pYPQ203 |
| 3382 | CG2 | pYPQ141-ZmUbi-RZ-CG2 | Pa2Cas12a | pYPQ203 |
| 3383 | CG2 | pYPQ141-ZmUbi-RZ-CG2 | Pc1Cas12a | pYPQ203 |
| 3384 | CG2 | pYPQ141-ZmUbi-RZ-CG2 | Sc1Cas12a | pYPQ203 |
| 3385 | CG2 | pYPQ141-ZmUbi-RZ-CG2 | LbCas12a | pYPQ203 |
| 3610 | CC1 | pYPQ141-ZmUbi-RZ-Fn-CC1 | LbCas12a | pYPQ203 |
| 3611 | CC1 | pYPQ141-ZmUbi-RZ-Fn-CC1 | Ba1Cas12 | pYPQ203 |
| 3612 | CC1 | pYPQ141-ZmUbi-RZ-Fn-CC1 | Ca1Cas12a | pYPQ203 |
| 3613 | CC1 | pYPQ141-ZmUbi-RZ-Fn-CC1 | Cb1Cas12a | pYPQ203 |
| 3614 | CC1 | pYPQ141-ZmUbi-RZ-Fn-CC1 | Cc1Cas12a | pYPQ203 |
| 3615 | CC1 | pYPQ141-ZmUbi-RZ-Fn-CC1 | Cu1Cas12a | pYPQ203 |
| 3616 | CC1 | pYPQ141-ZmUbi-RZ-Fn-CC1 | Ev1Cas12a | pYPQ203 |
| 3617 | CC1 | pYPQ141-ZmUbi-RZ-Fn-CC1 | Hs1Cas12a | pYPQ203 |
| 3618 | CC1 | pYPQ141-ZmUbi-RZ-Fn-CC1 | Pa2Cas12a | pYPQ203 |
| 3619 | CC1 | pYPQ141-ZmUbi-RZ-Fn-CC1 | Pc1Cas12a | pYPQ203 |
| 3620 | CC1 | pYPQ141-ZmUbi-RZ-Fn-CC1 | Sc1Cas12a | pYPQ203 |
| 3621 | GG1 | pYPQ141-ZmUbi-RZ-Fn-GG1 | LbCas12a | pYPQ203 |
| 3622 | GG1 | pYPQ141-ZmUbi-RZ-Fn-GG1 | Ba1Cas12 | pYPQ203 |
| 3623 | GG1 | pYPQ141-ZmUbi-RZ-Fn-GG1 | Ca1Cas12a | pYPQ203 |
| 3624 | GG1 | pYPQ141-ZmUbi-RZ-Fn-GG1 | Cb1Cas12a | pYPQ203 |
| 3625 | GG1 | pYPQ141-ZmUbi-RZ-Fn-GG1 | Cc1Cas12a | pYPQ203 |
| 3626 | GG1 | pYPQ141-ZmUbi-RZ-Fn-GG1 | Cu1Cas12a | pYPQ203 |
| 3627 | GG1 | pYPQ141-ZmUbi-RZ-Fn-GG1 | Ev1Cas12a | pYPQ203 |
| 3628 | GG1 | pYPQ141-ZmUbi-RZ-Fn-GG1 | Hs1Cas12a | pYPQ203 |
| 3629 | GG1 | pYPQ141-ZmUbi-RZ-Fn-GG1 | Pa2Cas12a | pYPQ203 |
| 3630 | GG1 | pYPQ141-ZmUbi-RZ-Fn-GG1 | Pc1Cas12a | pYPQ203 |
| 3631 | GG1 | pYPQ141-ZmUbi-RZ-Fn-GG1 | Sc1Cas12a | pYPQ203 |
| 3666 | SGR1-1,2;Blc;LCY-B1;LCY-B2;LCY-E | pYPQ146-35S-Lb-II | LbCas12a | pMDC32 |
| 3667 | SGR1-1,2;Blc;LCY-B1;LCY-B2;LCY-E | pYPQ146-35S-Lb-II | Mb2Cas12a | pMDC32 |
| 3869 | SGR1-1,2;Blc;LCY-B1;LCY-B2;LCY-E | pYPQ146-35S-Lb-II | Ev1Cas12a | pMDC32 |
| 3870 | SGR1-1,2;Blc;LCY-B1;LCY-B2;LCY-E | pYPQ146-35S-Lb-II | Hs1Cas12a | pMDC32 |
| 3871 | SGR1-1,2;Blc;LCY-B1;LCY-B2;LCY-E | pYPQ146-35S-Lb-II | Pc1Cas12a | pMDC32 |
| 4029 | L9,CC1,GG1,N28,N16,N20 | pYPQ146-ZmUbi-Mb2RVRR-6T | LbCas12a | pYPQ203 |
| 4030 | L9,CC1,GG1,N28,N16,N20 | pYPQ146-ZmUbi-Mb2RVRR-6T | Mb2Cas12a | pYPQ203 |
| 4031 | L9,CC1,GG1,N28,N16,N20 | pYPQ146-ZmUbi-Mb2RVRR-6T | Ev1Cas12a | pYPQ203 |
| 4032 | L9,CC1,GG1,N28,N16,N20 | pYPQ146-ZmUbi-Mb2RVRR-6T | Hs1Cas12a | pYPQ203 |
| 4351 | 4CL1-1,2,Pll1,2, SVP1,2 | pYPQ146-AtUBQ10-Pt-6crRNA-Lb | LbCas12a | pYPQ202 |
| 4355 | 4CL1-1,2,Pll1,2, SVP1,2 | pYPQ146-AtUBQ10-Pt-6crRNA-Fn | AsCas12a | pYPQ202 |
| 4356 | 4CL1-1,2,Pll1,2, SVP1,2 | pYPQ146-AtUBQ10-Pt-6crRNA-Fn | Mb2Cas12a | pYPQ202 |
| 4358 | 4CL1-1,2,Pll1,2, SVP1,2 | pYPQ146-AtUBQ10-Pt-6crRNA-Fn | Ev1Cas12a | pYPQ202 |
| 4359 | 4CL1-1,2,Pll1,2, SVP1,2 | pYPQ146-AtUBQ10-Pt-6crRNA-Fn | Hs1Cas12a | pYPQ202 |

Table S4. Oligos used in this study

| **Name** | **5'-3' sequence** | **Usage** |
| --- | --- | --- |
| 4CL1-crRNA1-F | TAGATGCAACCCTGTAGTCCCTGATGAA | crRNA cloning |
| 4CL1-crRNA1-R | GGCCTTCATCAGGGACTACAGGGTTGCA | crRNA cloning |
| 4CL1-crRNA2-F | TAGATCCAAGGAACCATTCGACATAAAA | crRNA cloning |
| 4CL1-crRNA2-R | GGCCTTTTATGTCGAATGGTTCCTTGGA | crRNA cloning |
| Pll-crRNA1-F | TAGATAAACCTCTCAAGTTGAAGAATTC | crRNA cloning |
| Pll-crRNA1-R | GGCCGAATTCTTCAACTTGAGAGGTTTA | crRNA cloning |
| Pll-crRNA2-F | TAGATAACCACCTTGAGCCCCAAAGCCT | crRNA cloning |
| Pll-crRNA2-R | GGCCAGGCTTTGGGGCTCAAGGTGGTTA | crRNA cloning |
| SVP-crRNA1-F | TAGATCACTCGAAGAATCTTGAGAAGCT | crRNA cloning |
| SVP-crRNA1-R | GGCCAGCTTCTCAAGATTCTTCGAGTGA | crRNA cloning |
| SVP-crRNA2-F | TAGATTCCGCAACTTCCTTACTCAACCT | crRNA cloning |
| SVP-crRNA2-R | GGCCAGGTTGAGTAAGGAAGTTGCGGAA | crRNA cloning |
| SlSGR1-crRNA1-F | TAGATgctatctcccaaaccatcaataa | crRNA cloning |
| SlSGR1-crRNA1-R | GGCCttattgatggtttgggagatagcA | crRNA cloning |
| SlSGR1-crRNA2-F | TAGATctagactcagaaactacatcttc | crRNA cloning |
| SlSGR1-crRNA2-R | GGCCgaagatgtagtttctgagtctagA | crRNA cloning |
| SlBlc-crRNA1-F | TAGATtgttaaagctaggagtagtactc | crRNA cloning |
| SlBlc-crRNA1-R | GGCCgagtactactcctagctttaacaA | crRNA cloning |
| SlLCY-B1-crRNA1-F | TAGATggtagaagtgtttgtgttaaggg | crRNA cloning |
| SlLCY-B1-crRNA1-R | GGCCcccttaacacaaacacttctaccA | crRNA cloning |
| SlLCY-B2-crRNA1-F | TAGATatgtgggtgctaaatcaagaaag | crRNA cloning |
| SlLCY-B2-crRNA1-R | GGCCctttcttgatttagcacccacatA | crRNA cloning |
| SlLCY-E-crRNA1-F | TAGATgcgggataccattgtatatcttg | crRNA cloning |
| SlLCY-E-crRNA1-R | GGCCcaagatatacaatggtatcccgcA | crRNA cloning |
| Hi-TOM-F-1 | ACACTCTTTCCCTACACGACGCTCTTCCGATCTgcttGCGTtggagtgagtacggtgtgc | Hi-TOM barcoding primer |
| Hi-TOM-F-2 | ACACTCTTTCCCTACACGACGCTCTTCCGATCTgcttGTAGtggagtgagtacggtgtgc | Hi-TOM barcoding primer |
| Hi-TOM-F-3 | ACACTCTTTCCCTACACGACGCTCTTCCGATCTgcttACGCtggagtgagtacggtgtgc | Hi-TOM barcoding primer |
| Hi-TOM-F-4 | ACACTCTTTCCCTACACGACGCTCTTCCGATCTgcttCTCGtggagtgagtacggtgtgc | Hi-TOM barcoding primer |
| Hi-TOM-F-5 | ACACTCTTTCCCTACACGACGCTCTTCCGATCTgcttGCTCtggagtgagtacggtgtgc | Hi-TOM barcoding primer |
| Hi-TOM-F-6 | ACACTCTTTCCCTACACGACGCTCTTCCGATCTgcttAGTCtggagtgagtacggtgtgc | Hi-TOM barcoding primer |
| Hi-TOM-F-7 | ACACTCTTTCCCTACACGACGCTCTTCCGATCTgcttCGACtggagtgagtacggtgtgc | Hi-TOM barcoding primer |
| Hi-TOM-F-8 | ACACTCTTTCCCTACACGACGCTCTTCCGATCTgcttGATGtggagtgagtacggtgtgc | Hi-TOM barcoding primer |
| Hi-TOM-F-9 | ACACTCTTTCCCTACACGACGCTCTTCCGATCTgcttATACtggagtgagtacggtgtgc | Hi-TOM barcoding primer |
| Hi-TOM-F-10 | ACACTCTTTCCCTACACGACGCTCTTCCGATCTgcttCACAtggagtgagtacggtgtgc | Hi-TOM barcoding primer |
| Hi-TOM-F-11 | ACACTCTTTCCCTACACGACGCTCTTCCGATCTgcttGTGCtggagtgagtacggtgtgc | Hi-TOM barcoding primer |
| Hi-TOM-F-12 | ACACTCTTTCCCTACACGACGCTCTTCCGATCTgcttACTAtggagtgagtacggtgtgc | Hi-TOM barcoding primer |
| Hi-TOM-R-A | GACTGGAGTTCAGACGTGTGCTCTTCCGATCTctgtGCGTtgagttggatgctggatgg | Hi-TOM barcoding primer |
| Hi-TOM-R-B | GACTGGAGTTCAGACGTGTGCTCTTCCGATCTctgtGTAGtgagttggatgctggatgg | Hi-TOM barcoding primer |
| Hi-TOM-R-C | GACTGGAGTTCAGACGTGTGCTCTTCCGATCTctgtACGCtgagttggatgctggatgg | Hi-TOM barcoding primer |
| Hi-TOM-R-D | GACTGGAGTTCAGACGTGTGCTCTTCCGATCTctgtCTCGtgagttggatgctggatgg | Hi-TOM barcoding primer |
| Hi-TOM-R-E | GACTGGAGTTCAGACGTGTGCTCTTCCGATCTctgtGCTCtgagttggatgctggatgg | Hi-TOM barcoding primer |
| Hi-TOM-R-F | GACTGGAGTTCAGACGTGTGCTCTTCCGATCTctgtAGTCtgagttggatgctggatgg | Hi-TOM barcoding primer |
| Hi-TOM-R-G | GACTGGAGTTCAGACGTGTGCTCTTCCGATCTctgtCGACtgagttggatgctggatgg | Hi-TOM barcoding primer |
| Hi-TOM-R-H | GACTGGAGTTCAGACGTGTGCTCTTCCGATCTctgtGATGtgagttggatgctggatgg | Hi-TOM barcoding primer |
| 4CL1-Hitom-F3 | ggagtgagtacggtgtgcGGGTGCTTGCACTTTTCAGA | Hi-TOM barcoding primer |
| 4CL1-Hitom-R3 | gagttggatgctggatggCTCCATCTACCTGTTGAGCCA | Hi-TOM barcoding primer |
| 4CL1-Hitom-F4 | ggagtgagtacggtgtgcGGGATATGGAATGACCGAGG | Hi-TOM barcoding primer |
| 4CL1-Hitom-R4 | gagttggatgctggatggTCATGATCTGATCACCCCGG | Hi-TOM barcoding primer |
| PII-Hitom-F1 | ggagtgagtacggtgtgcAAACCAGGCTCCCTCACTTC | Hi-TOM barcoding primer |
| PII-Hitom-R1 | gagttggatgctggatggCGCATACCAGGAACAGAGGA | Hi-TOM barcoding primer |
| PII-Hitom-F2 | ggagtgagtacggtgtgcGGCTCTGTTGAAAATTGGTATTCG | Hi-TOM barcoding primer |
| PII-Hitom-R2 | gagttggatgctggatggGCGAAAGGAGACGAAACAGAA | Hi-TOM barcoding primer |
| SVP-Hitom-F1 | ggagtgagtacggtgtgcCTTCCAGGCCATCTTAGGAAATGA | Hi-TOM barcoding primer |
| SVP-Hitom-R1 | gagttggatgctggatggGCACTGTCTTAAACACTCTCCAC | Hi-TOM barcoding primer |
| SVP-Hitom-F2 | ggagtgagtacggtgtgcCCTGACTTCTTTTACATTAGCTGTGG | Hi-TOM barcoding primer |
| SVP-Hitom-R2 | gagttggatgctggatggAGGCATAGAGCTGCTAGAAGC | Hi-TOM barcoding primer |
| Hi-TOM-CG2-F | ggagtgagtacggtgtgcAAGACAGATTCCTCTGCATTGA | Hi-TOM barcoding primer |
| Hi-TOM-CG2-R | gagttggatgctggatggGCGTCAAACTTTCCTTAACAGG | Hi-TOM barcoding primer |
| Hi-TOM-CC1-F | ggagtgagtacggtgtgcGCCAAAAGTGTATGAGACAAAGG | Hi-TOM barcoding primer |
| Hi-TOM-CC1-R | gagttggatgctggatggGCTGGTGCCACTGTTGAATA | Hi-TOM barcoding primer |
| Hi-TOM-GG1-F | ggagtgagtacggtgtgcTATAAGCCACCGATCCCACA | Hi-TOM barcoding primer |
| Hi-TOM-GG1-R | gagttggatgctggatggGCATTATTGAAGGCTCGTTCC | Hi-TOM barcoding primer |
| CC1-TTV-HiTom-F1 | gagttggatgctggatggCGAATCACGTGTACATCACA | Hi-TOM barcoding primer |
| CC1-TTV HiTom R1 | gagttggatgctggatggGACTACAGAAACAAGGTGC | Hi-TOM barcoding primer |
| GG1-TTV-HiTom-F1 | ggagtgagtacggtgtgcCTGACCCAACAACAGCCAAA | Hi-TOM barcoding primer |
| GG1-TTV-HiTom-R2 | gagttggatgctggatggGCATCATTTAGCCAGGGATC | Hi-TOM barcoding primer |
