# Supplementary material for: Hs1Cas12a and Ev1Cas12a confer efficient genome editing in plants
Source: Front Genome Ed. 2023 Oct 12;5:1251903. doi: 10.3389/fgeed.2023.1251903 (PMC10602648; doi:10.3389/fgeed.2023.1251903)
Supplement: Supplementary file 2 [file Presentation1.PPTX]

## Slide 1
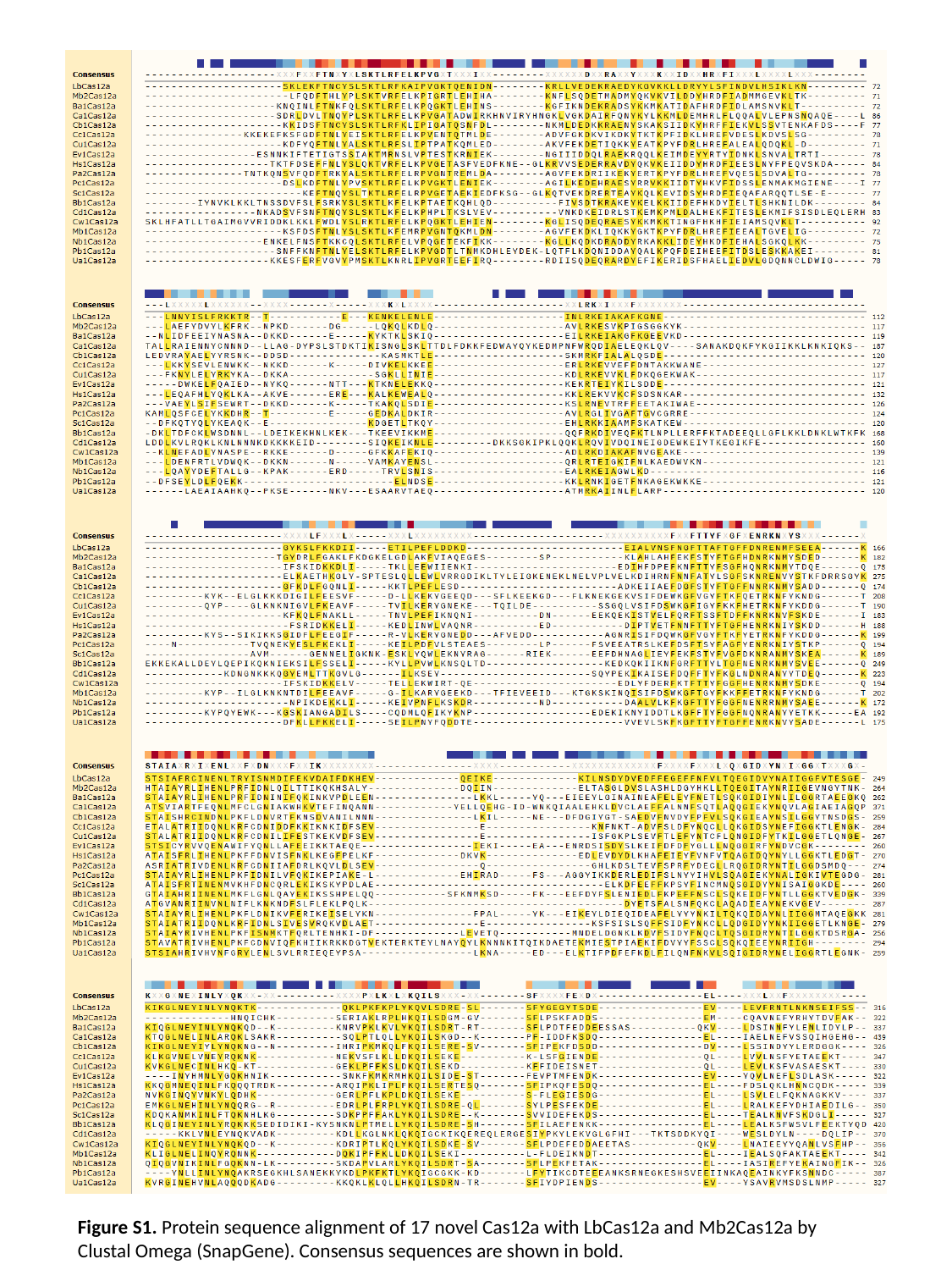

Figure S1. Protein sequence alignment of 17 novel Cas12a with LbCas12a and Mb2Cas12a by Clustal Omega (SnapGene). Consensus sequences are shown in bold.

## Slide 2
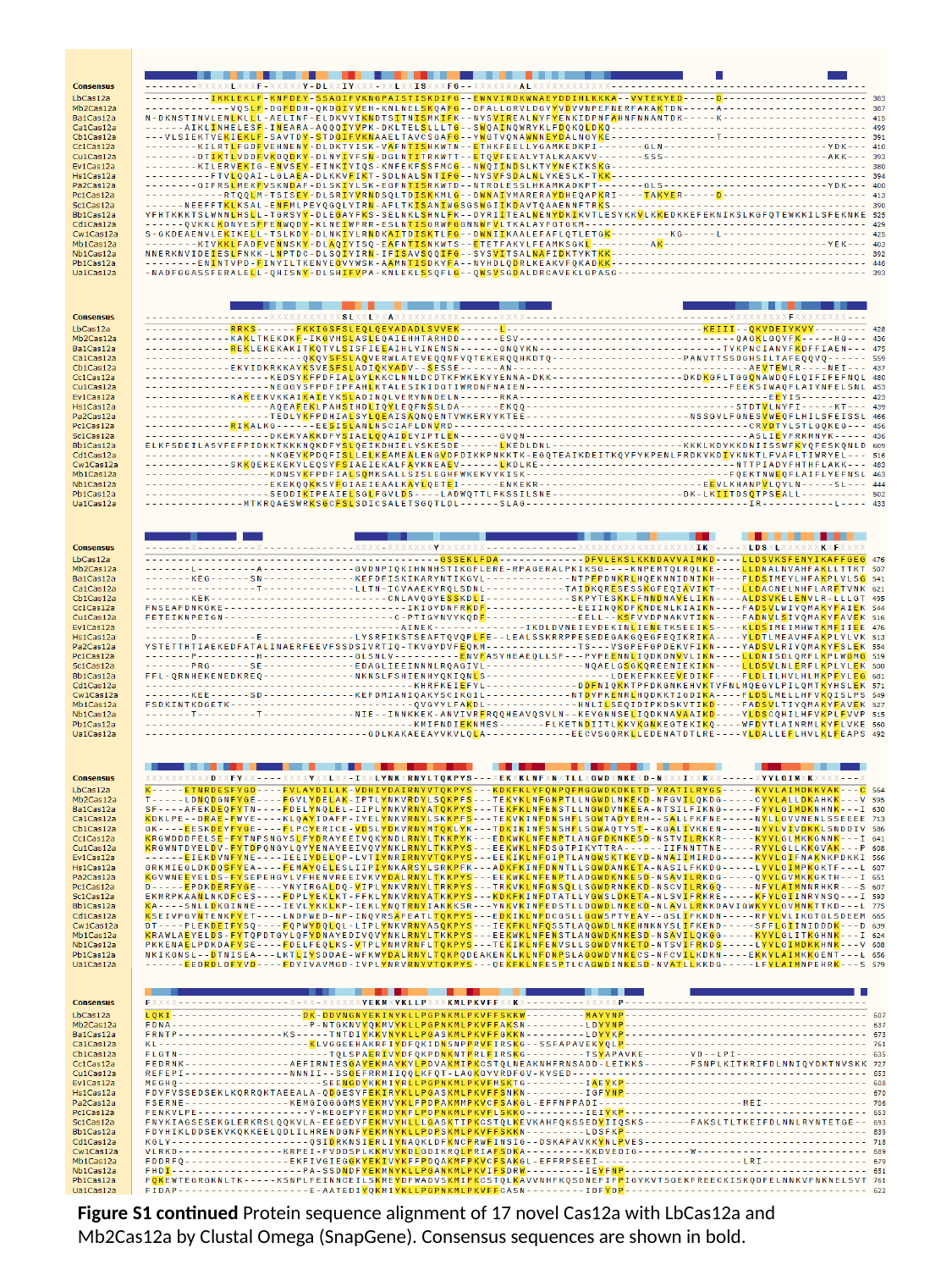

Figure S1 continued Protein sequence alignment of 17 novel Cas12a with LbCas12a and Mb2Cas12a by Clustal Omega (SnapGene). Consensus sequences are shown in bold.

## Slide 3
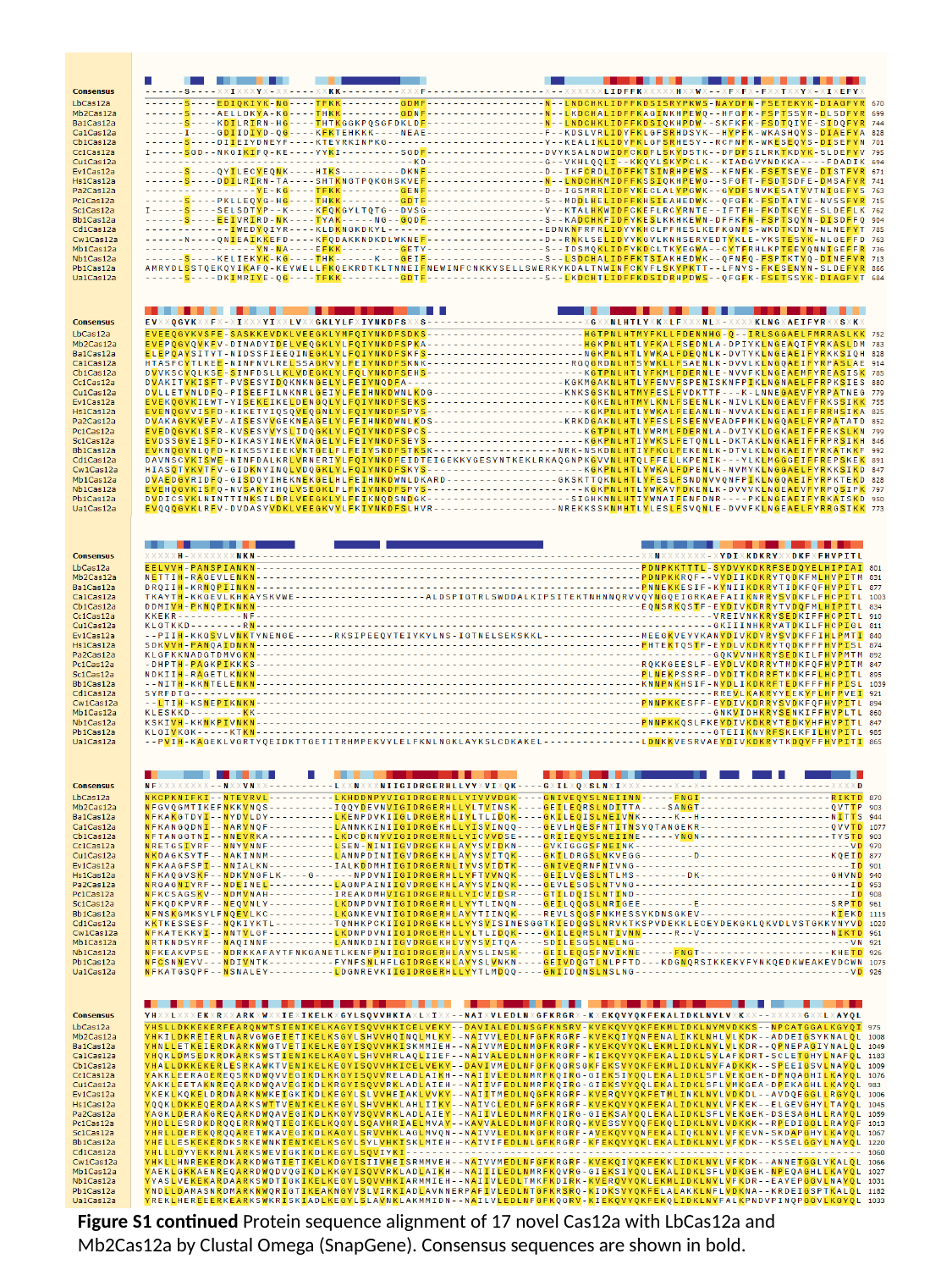

Figure S1 continued Protein sequence alignment of 17 novel Cas12a with LbCas12a and Mb2Cas12a by Clustal Omega (SnapGene). Consensus sequences are shown in bold.

## Slide 4
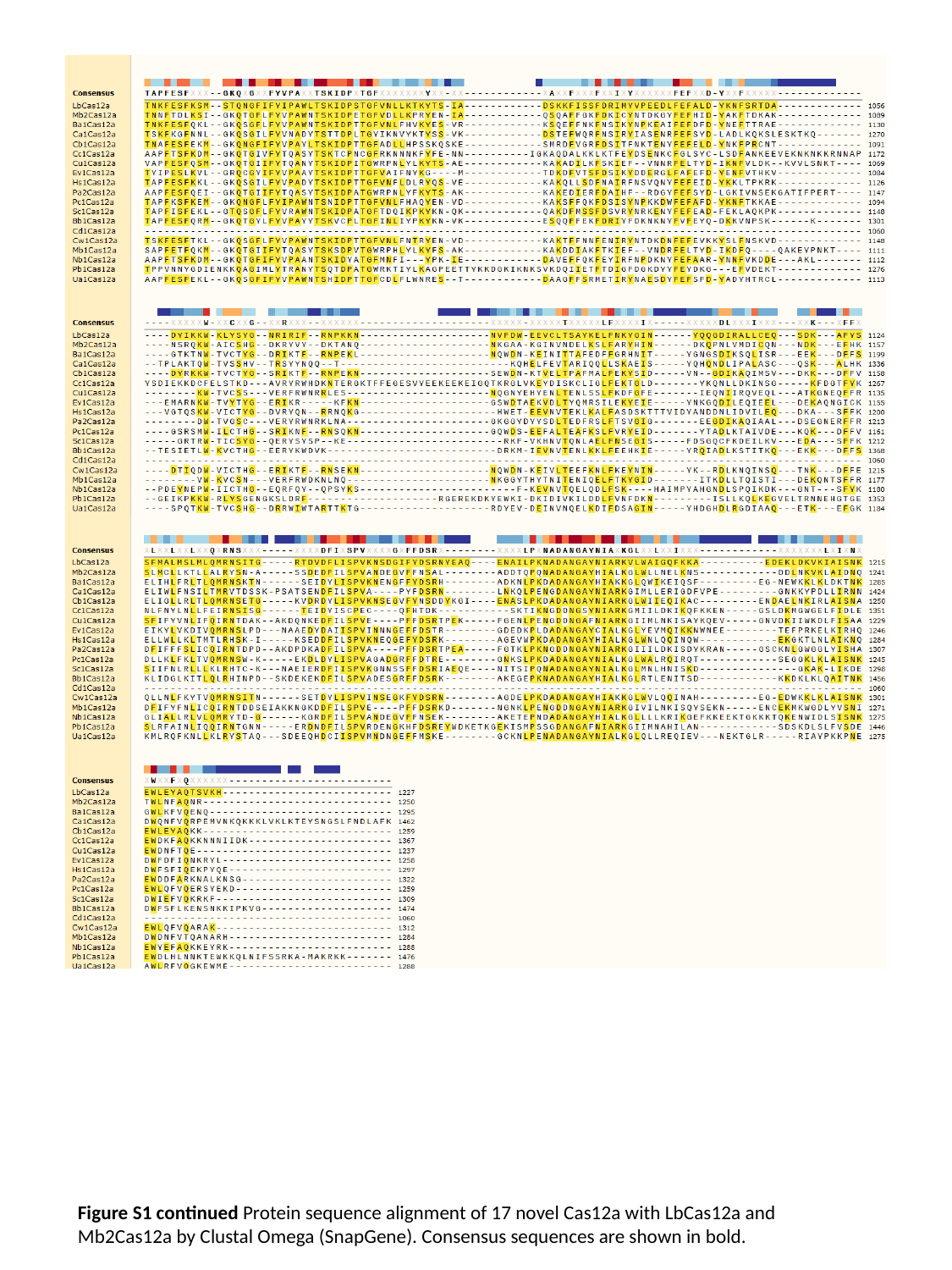

Figure S1 continued Protein sequence alignment of 17 novel Cas12a with LbCas12a and Mb2Cas12a by Clustal Omega (SnapGene). Consensus sequences are shown in bold.

## Slide 5
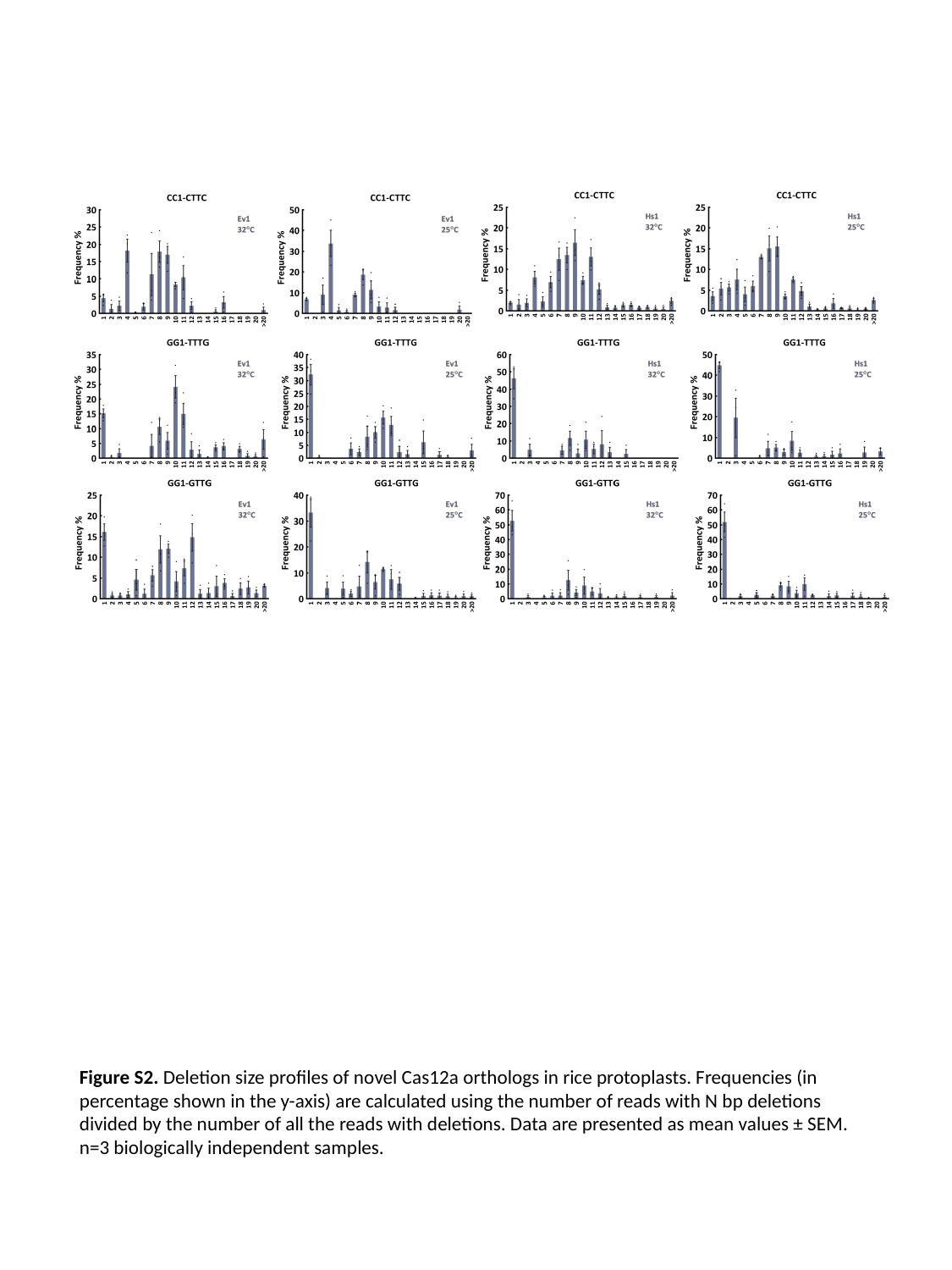

Figure S2. Deletion size profiles of novel Cas12a orthologs in rice protoplasts. Frequencies (in percentage shown in the y-axis) are calculated using the number of reads with N bp deletions divided by the number of all the reads with deletions. Data are presented as mean values ± SEM. n=3 biologically independent samples.

## Slide 6
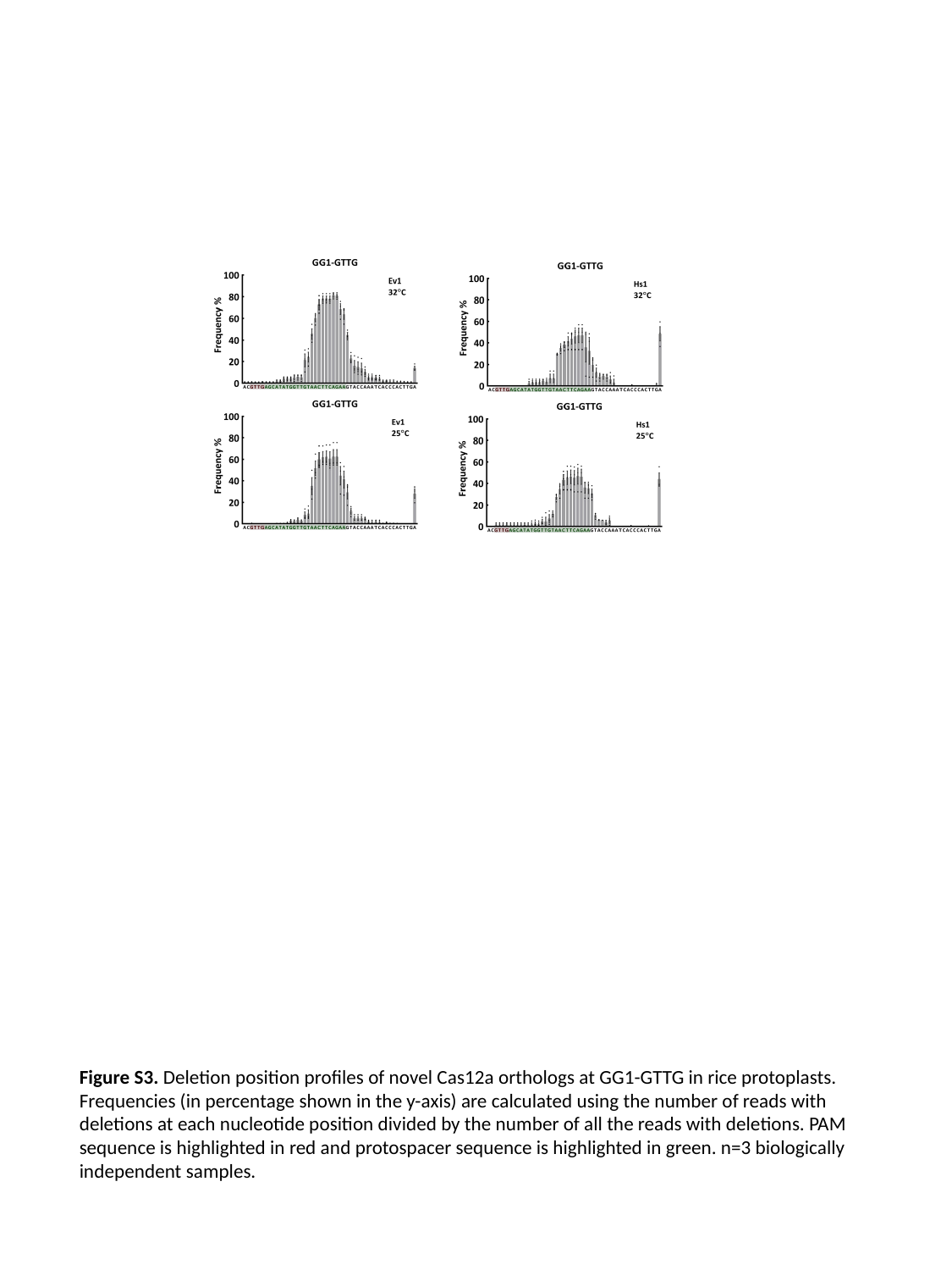

Figure S3. Deletion position profiles of novel Cas12a orthologs at GG1-GTTG in rice protoplasts. Frequencies (in percentage shown in the y-axis) are calculated using the number of reads with deletions at each nucleotide position divided by the number of all the reads with deletions. PAM sequence is highlighted in red and protospacer sequence is highlighted in green. n=3 biologically independent samples.

## Slide 7
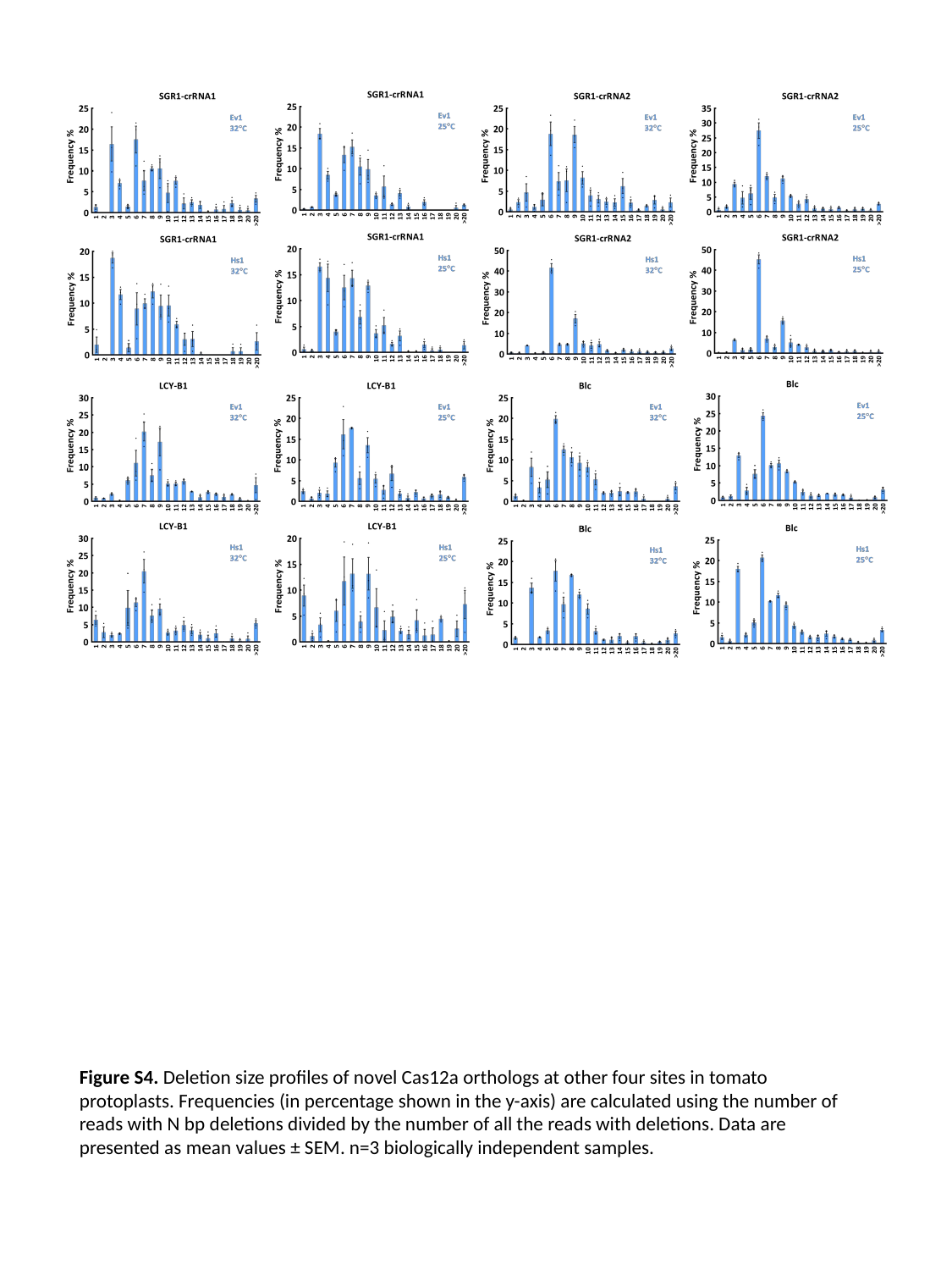

Figure S4. Deletion size profiles of novel Cas12a orthologs at other four sites in tomato protoplasts. Frequencies (in percentage shown in the y-axis) are calculated using the number of reads with N bp deletions divided by the number of all the reads with deletions. Data are presented as mean values ± SEM. n=3 biologically independent samples.

## Slide 8
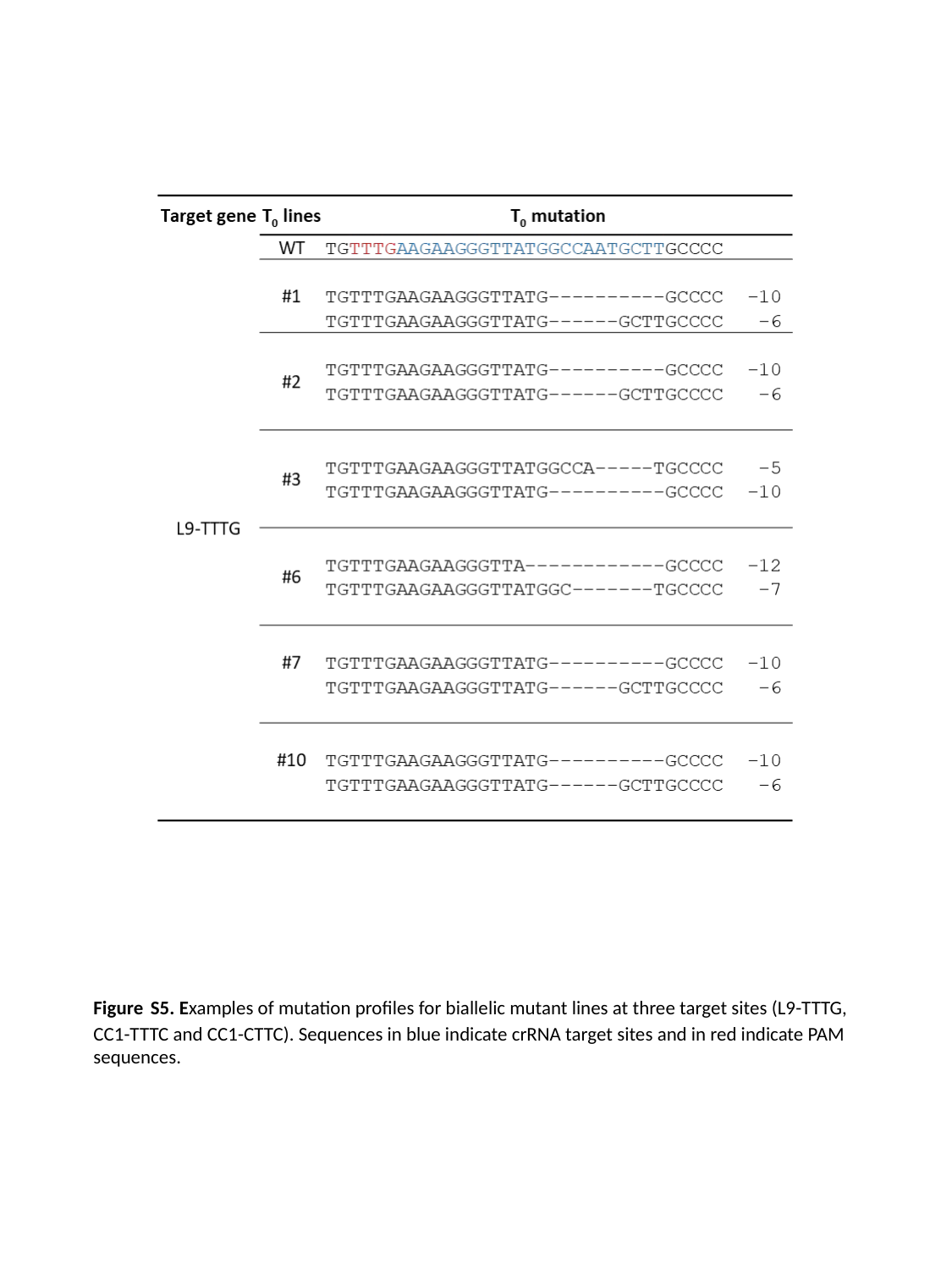

Figure S5. Examples of mutation profiles for biallelic mutant lines at three target sites (L9-TTTG, CC1-TTTC and CC1-CTTC). Sequences in blue indicate crRNA target sites and in red indicate PAM sequences.

## Slide 9
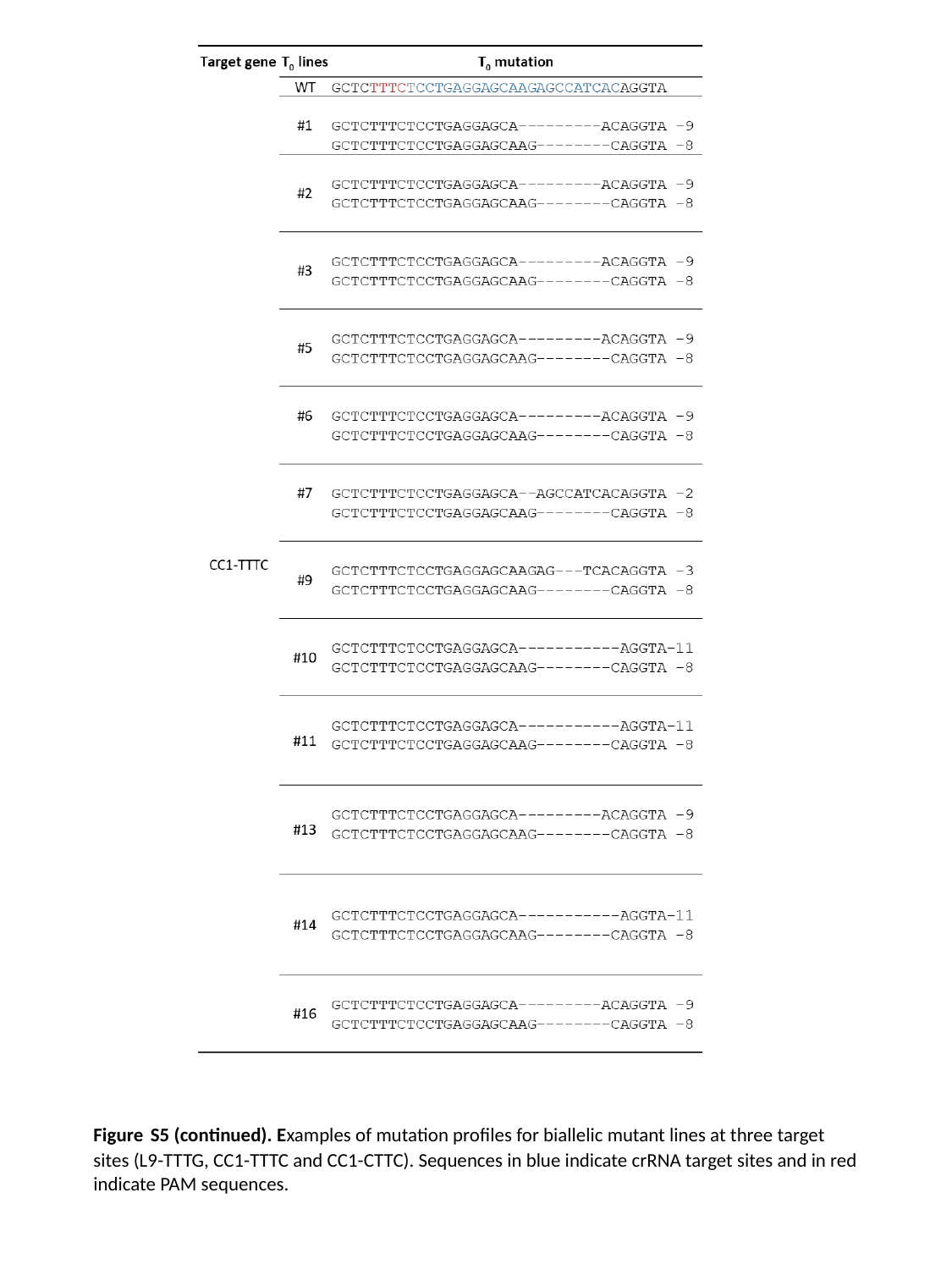

Figure S5 (continued). Examples of mutation profiles for biallelic mutant lines at three target sites (L9-TTTG, CC1-TTTC and CC1-CTTC). Sequences in blue indicate crRNA target sites and in red indicate PAM sequences.

## Slide 10
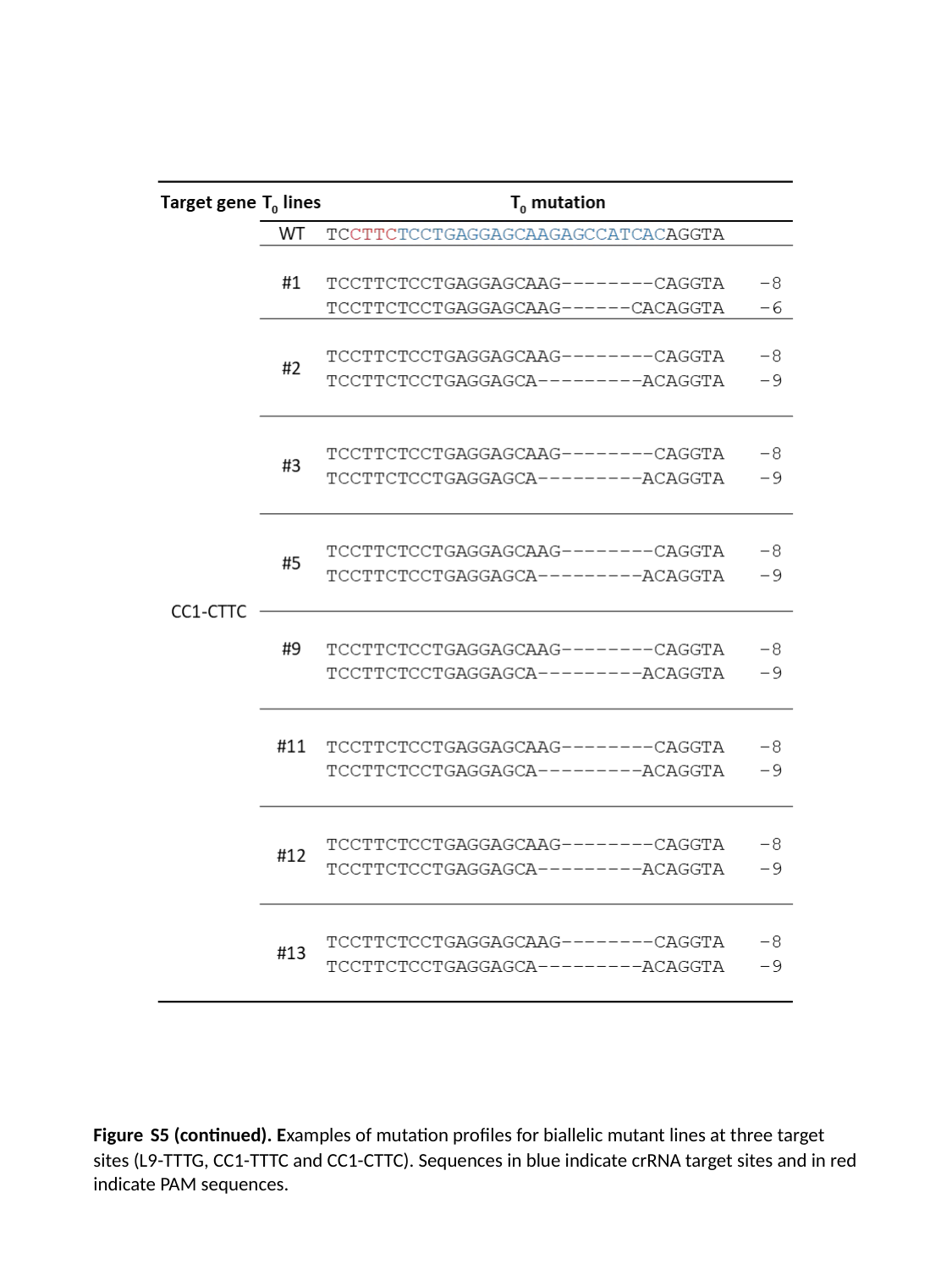

Figure S5 (continued). Examples of mutation profiles for biallelic mutant lines at three target sites (L9-TTTG, CC1-TTTC and CC1-CTTC). Sequences in blue indicate crRNA target sites and in red indicate PAM sequences.

## Slide 11
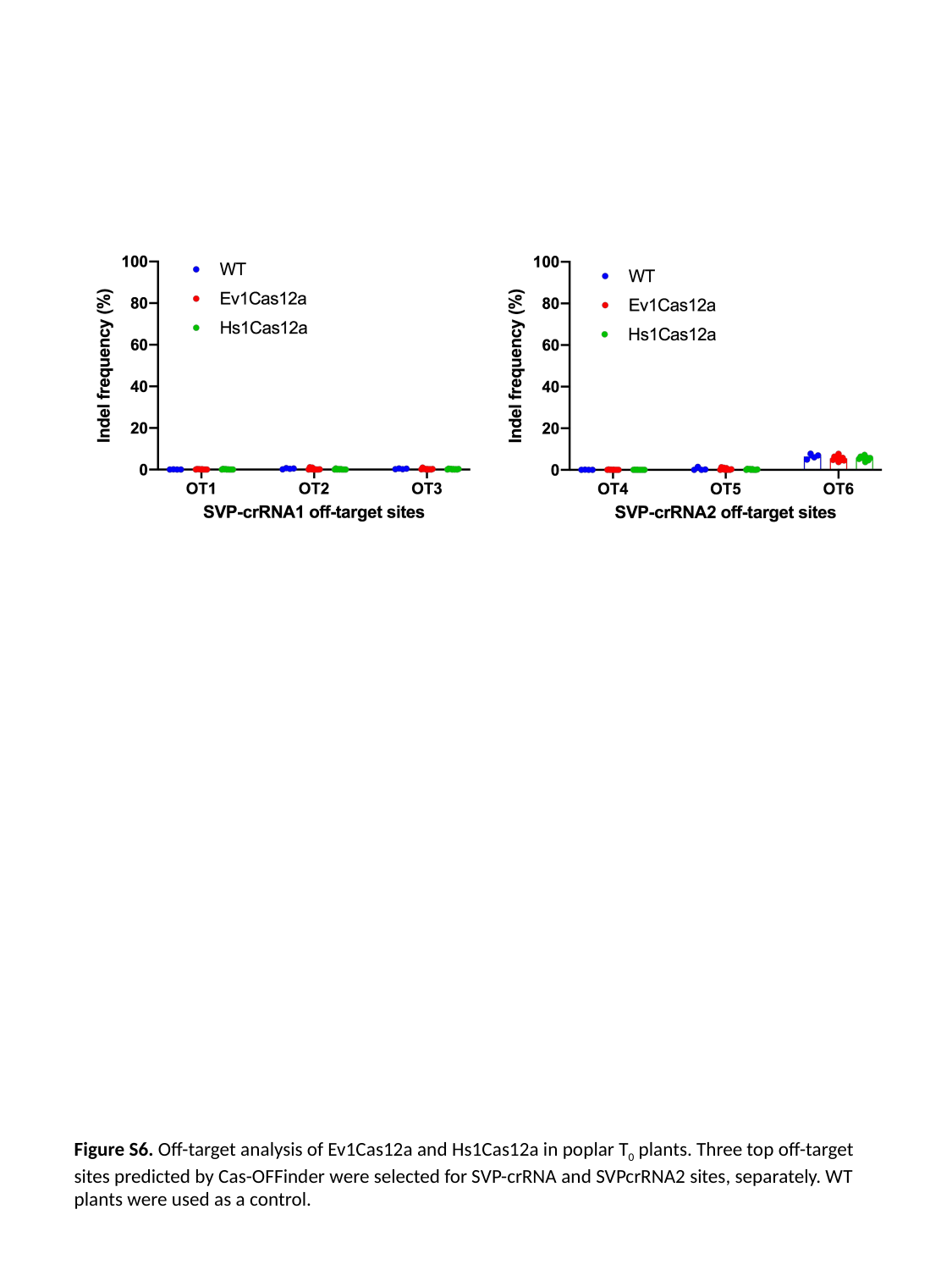

Figure S6. Off-target analysis of Ev1Cas12a and Hs1Cas12a in poplar T0 plants. Three top off-target sites predicted by Cas-OFFinder were selected for SVP-crRNA and SVPcrRNA2 sites, separately. WT plants were used as a control.

## Slide 12
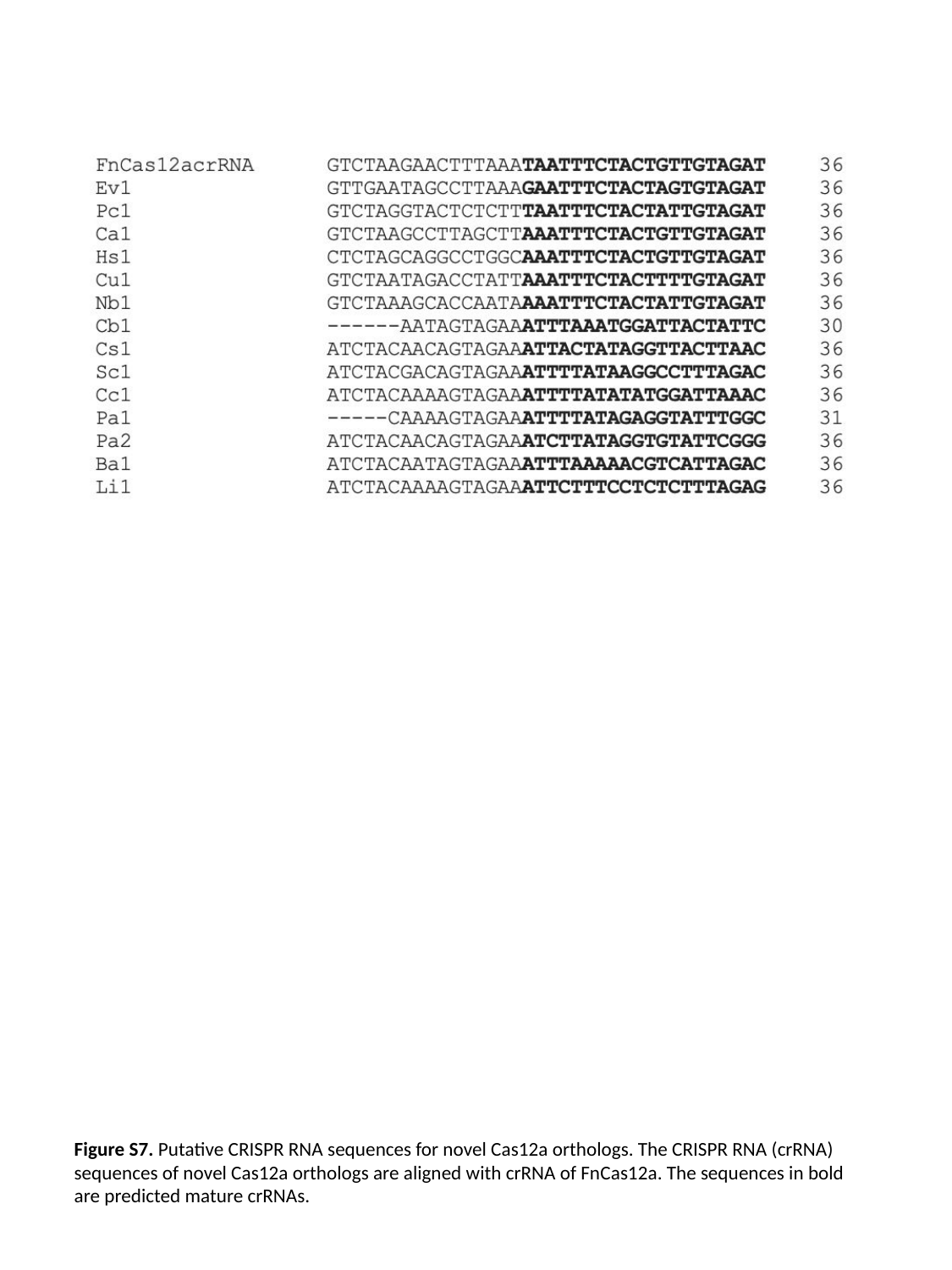

Figure S7. Putative CRISPR RNA sequences for novel Cas12a orthologs. The CRISPR RNA (crRNA) sequences of novel Cas12a orthologs are aligned with crRNA of FnCas12a. The sequences in bold are predicted mature crRNAs.

## Slide 13
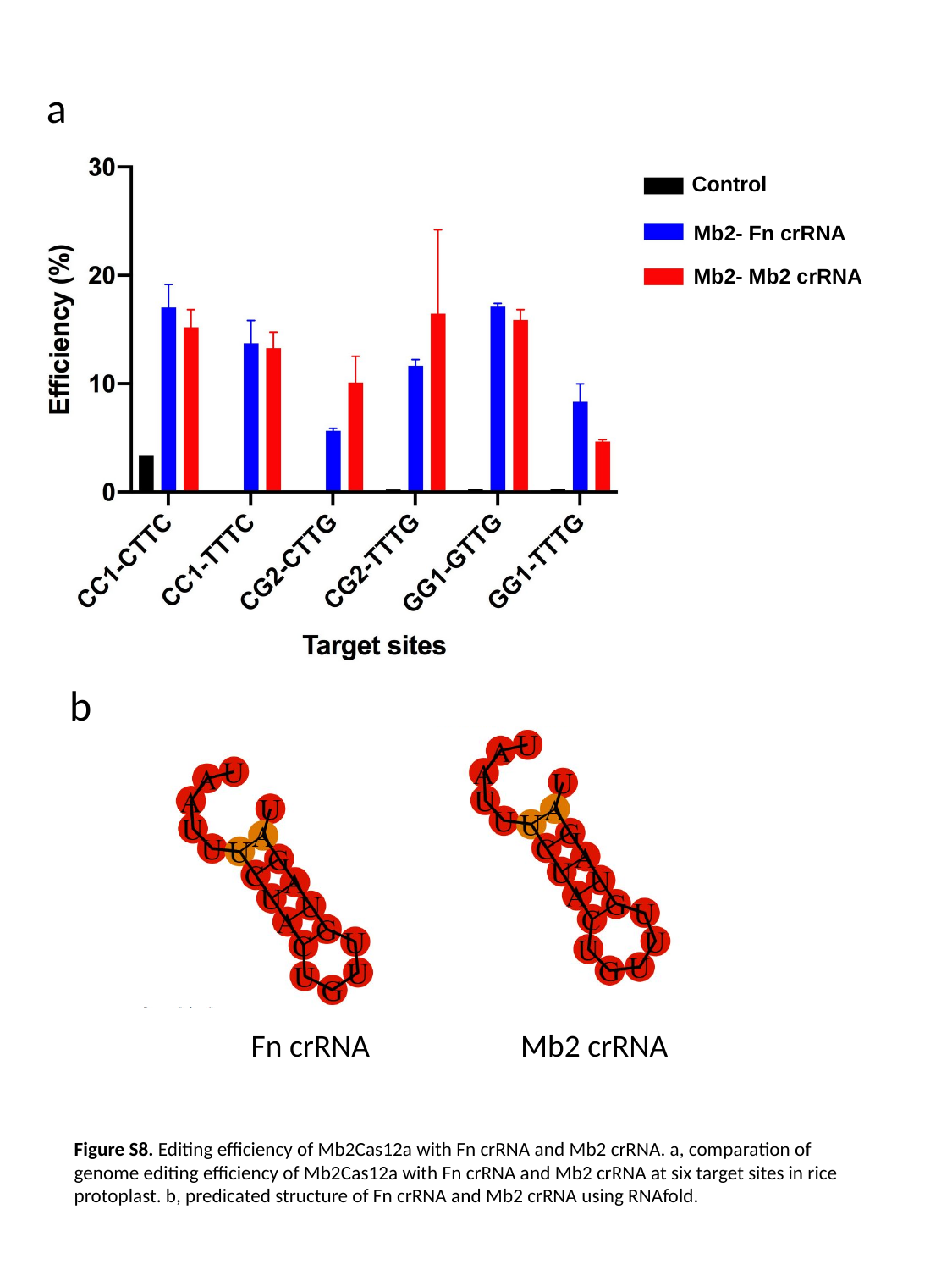

a
Control
Mb2- Fn crRNA
Mb2- Mb2 crRNA
b
Fn crRNA
Mb2 crRNA
Figure S8. Editing efficiency of Mb2Cas12a with Fn crRNA and Mb2 crRNA. a, comparation of genome editing efficiency of Mb2Cas12a with Fn crRNA and Mb2 crRNA at six target sites in rice protoplast. b, predicated structure of Fn crRNA and Mb2 crRNA using RNAfold.
